# A relay race of ESCRT-III paralogs drives cell division in a hyperthermophilic archaeon

**DOI:** 10.1128/mbio.00991-24

**Published:** 2024-12-19

**Authors:** Junfeng Liu, Mickaël Lelek, Yunfeng Yang, Audrey Salles, Christophe Zimmer, Yulong Shen, Mart Krupovic

**Affiliations:** 1 Institut Pasteur, Université Paris Cité, CNRS UMR6047, Archaeal Virology Unit, Paris, France; 2 CRISPR and Archaea Biology Research Centre, Microbial Technology Institute and State Key Laboratory of Microbial Technology, Shandong University, Qingdao, China; 3 Imaging and Modeling Unit, Institut Pasteur, Université Paris Cité, Paris, France; 4 Institut Pasteur, Université Paris Cité, Unit of Technology and Service Photonic BioImaging (UTechS PBI), C2RT, Paris, France; 5 Rudolf Virchow Center, University of Würzburg, Würzburg, Germany; University of Vienna, Vienna, Austria

**Keywords:** *Archaea*, cell division, ESCRT system, *Crenarchaeota*, *Sulfolobus*, *Saccharolobus islandicus*

## Abstract

Cell division is a fundamental process ensuring the perpetuation of all cellular life forms. Archaea of the order Sulfolobales divide using a simpler version of the eukaryotic endosomal sorting complexes required for transport (ESCRT) machinery, composed of three ESCRT-III homologs (ESCRT-III, -III-1, and -III-2), AAA+ ATPase Vps4 and an archaea-specific component CdvA. Here, we clarify how these components act sequentially to drive the division of the hyperthermophilic archaeon *Saccharolobus islandicus*. Our data suggest that ESCRT-III plays an active role during the early stage of membrane constriction during cytokinesis, whereas ESCRT-III-1 and ESCRT-III-2 are indispensable for the “pre-late” and “late” stages of cytokinesis, respectively. In the *escrt-III-1* deletion strain, the division is blocked when the mid-cell constriction reaches ~30% of the initial cell diameter (“pre-late” stage), yielding “chain-like” cellular aggregates. Depletion of ESCRT-III-2 leads to the accumulation of cells connected through narrow membrane bridges (“late” stage), consistent with the key role of this protein in the final membrane abscission. We used 3D-single molecule localization microscopy to image ESCRT-III rings of different diameters and show that the decrease in the ESCRT-III ring diameter and membrane constriction are inconsistent with a mechanism exclusively based on spiraling of the ESCRT-III filaments. By contrast, the cone-shaped assemblies of ESCRT-III-1 and ESCRT-III-2 are consistent with spiral formation, highlighting the distinct roles of the three ESCRT-III proteins during the cytokinesis. We propose the “relay race” model, whereby the cytokinesis is achieved through a sequential and concerted action of different ESCRT machinery components.

**IMPORTANCE:**

Two major cytokinesis mechanisms, rooted in contractile FtsZ and endosomal sorting complexes required for transport (ESCRT) rings, respectively, have emerged in the course of evolution. Whereas bacteria rely on the FtsZ-based mechanism, different lineages of archaea use either of the two systems, and eukaryotes have inherited the ESCRT-based cell division machinery from their archaeal ancestors. The mechanism of ESCRT-based cell division in archaea remains poorly understood and mechanistic studies on different archaeal model systems are essential to unravel the natural history of the ESCRT machinery. Here we investigate the interplay between three major ESCRT-III homologs during the division of a hyperthermophilic archaeon *Saccharolobus islandicus* and propose the “relay race” model of cytokinesis.

## INTRODUCTION

Cell division is a fundamental process underlying the perpetuation of cellular life on Earth. Two non-homologous cell division machineries based on contractile FtsZ and endosomal sorting complexes required for transport (ESCRT) rings, respectively, have emerged during evolution. Bacteria divide using the FtsZ-based system (1), whereas in eukaryotes, membrane abscission during cytokinesis is mediated by the ESCRT machinery (2, 3). The ESCRT system can be subdivided into several functionally distinct subcomplexes known as ESCRT-0, ESCRT-I, ESCRT-II, and ESCRT-III, and the AAA+ ATPase Vps4, but only ESCRT-III and Vps4 are universally involved in ESCRT-dependent membrane remodeling. Multiple homologs of ESCRT-III form membrane-associated filaments, whereas the Vps4 ATPase binds directly to ESCRT-III filaments and disassembles them in an ATP-dependent manner, driving membrane deformation (4–6).

Members of the domain Archaea display dichotomous distribution of cell division machineries, with some archaea using the bacterial-like FtsZ-based system and others dividing using the eukaryotic-like ESCRT-based machinery (7). The majority of archaea from the Thermoproteota and Asgardarchaeota phyla encode the ESCRT system (8–10), with Asgard archaea encoding ESCRT homologs that are phylogenetically more closely related to the eukaryotic ones (10–13). However, Asgard archaea remain extremely challenging to cultivate under laboratory conditions and lack genetic tools; hence, the role of their ESCRT machinery in membrane remodeling remains to be investigated *in vivo*. By contrast, genetically tractable hyperthermophilic archaea of the order Sulfolobales, in particular, members of the genera *Sulfolobus* and *Saccharolobus*, emerged as powerful models for elucidation of the role and functioning of the archaeal ESCRT machinery (9, 14–18).

In *Sulfolobus* and *Saccharolobus*, the ESCRT machinery consists of archaea-specific protein CdvA, four ESCRT-III homologs, namely, ESCRT-III (also known as CdvB), ESCRT-III-1 (CdvB1), ESCRT-III-2 (CdvB2), and ESCRT-III-3 (CdvB3), and the AAA+ ATPase Vps4 (CdvC) (16, 18–20). CdvA interacts with the membrane and initiates cell division by recruiting ESCRT-III homologs to the mid-cell, where they form ring-like structures on the membrane (21, 22). Subsequently, the expression of CdvA is repressed by a transcription factor aCcr1 until the next round of cell division (23). A recent study in *Sulfolobus acidocaldarius* showed that ESCRT-III (CdvB) forms a non-contractile ring at the mid-cell and acts as a template for recruitment of ESCRT-III-1 (CdvB1) and ESCRT-III-2 (CdvB2) which assemble into ring-like structures (24). Subsequent proteasome-mediated degradation of the static ESCRT-III ring was suggested to release the tension stored in the ESCRT-III-1 and ESCRT-III-2 polymers, driving membrane constriction and eventual cell division (16). Similar to eukaryotes, archaeal ESCRT machinery plays additional roles during various processes involving membrane remodeling, such as asymmetric division of virus-infected cells in *Saccharolobus islandicus* REY15A (25) and biogenesis of extracellular vesicle (24, 26). Although the general picture of ESCRT-mediated cell division in archaea is starting to emerge (27, 28), the mechanistic details of the interplay between different components of the ESCRT machinery remain poorly understood. In particular, it remains to be determined how different ESCRT-III homologs interact to achieve membrane constriction. Furthermore, it is unclear how Vps4 powers this process.

Here, we investigated the cell division process in *S. islandicus* REY15A using a collection of knockout and knockdown strains and strains expressing dominant-negative mutants of different components of the ESCRT machinery. We suggest that ESCRT-III of REY15A forms a contractile ring and plays a crucial role during the initial stages of membrane constriction. The interaction between ESCRT-III and CdvA was necessary for the ESCRT-III ring formation. Furthermore, our data suggests that the AAA+ ATPase Vps4 is necessary for the disassembly of the ESCRT-III ring prior to the proteasomal degradation of the ESCRT-III subunits. Expression of a dominant-negative mutant of Vps4 in different genetic backgrounds resulted in the arrest of cell division. Unexpectedly, ESCRT-III-1 and ESCRT-III-2 appeared to primarily function during the “pre-late” and “late” stages of cytokinesis, respectively, whereby small diameter membrane tubes connecting the two daughter cells are abscised. Finally, we used 3D super-resolution by single-molecule localization microscopy (SMLM) to analyze ESCRT-III rings with different diameters, which provided further insights into the constriction mechanism by ruling out the spiraling of the ESCRT-III ring upon constriction. Collectively, our results provide new insights into the division of *Saccharolobus* cells, clarifying the roles of different ESCRT machinery components.

## RESULTS

### ESCRT-III forms a contractile rather than static ring during cell division

**Fig 1:**
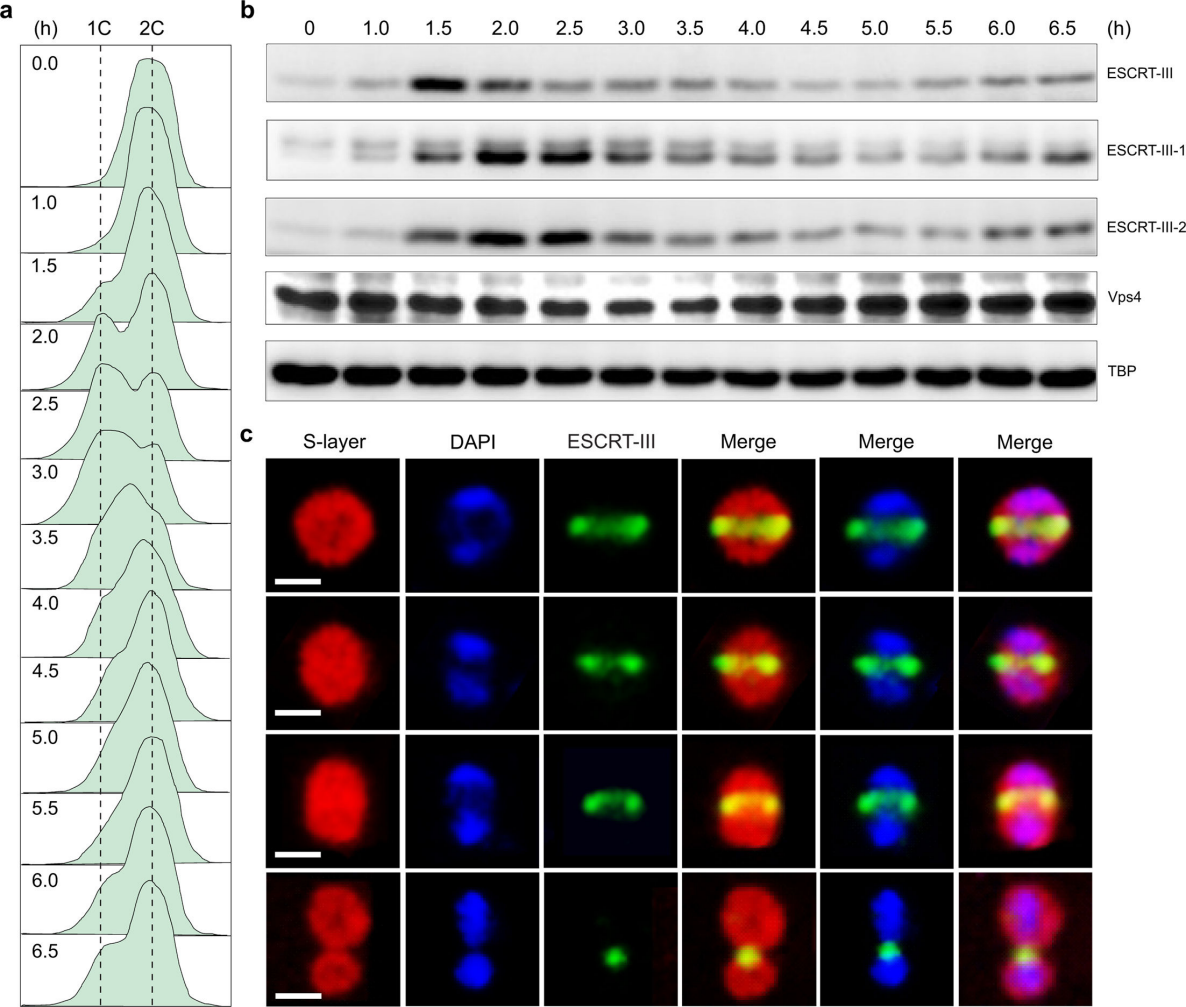
ESCRT-III exhibits a cyclic pattern and forms a contractile ring in synchronized *S. islandicus* REY15A cells. (a) Flow cytometry analysis of synchronized REY15A cells. The cells were synchronized to the G2 phase by acetic acid treatment. Cell division took place 1.5 h following the removal of acetic acid and the second round of division started at around 6 h. **(b)** Western blot analysis of the expression of the ESCRT machinery proteins in synchronized REY15A cells. The expression level of ESCRT-III peaked at 1.5 h following the removal of acetic acid, coinciding with the time when the cells started to divide and then declined. By contrast, the expression levels of ESCRT-III-1 and ESCRT-III-2 peaked at about 2.0 h. TATA-binding protein (TBP) was used as the loading control. Representative images are shown. **(c)** Confocal imaging of ESCRT-III in synchronized REY15A cells. The cells were collected 2 h after the removal of acetic acid and subjected to immunofluorescence microscopy. The representative images depict different stages of cell division. Nucleoids and proteinaceous surface (S-) layer were stained with 4’,6-diamidino-2-phenylindole (DAPI), and Alexa Fluor 647 conjugated to Concanavalin A, respectively. ESCRT-III was labeled with fluorescently labeled antibodies. Scale bars, 1 µm.

To study the cell division process in *Saccharolobus islandicus* strain REY15A, we synchronized the population by arresting the cell cycle using acetic acid treatment (26), which presumably induces starvation responses due to respiration uncoupling. As a result, *S. islandicus* cells are arrested in the post-replicative G2 phase of the cell cycle, characterized by the presence of two chromosome copies per cell (Fig. 1a). Removal of acetic acid (defined as 0 h timepoint) lifted the cell cycle arrest, with the cells starting to divide 1.5 h later; the second round of cell division took place at ~6.0 h (Fig. 1a). The levels of ESCRT-III, ESCRT-III-1, and ESCRT-III-2 all displayed cyclical patterns, with the level of ESCRT-III peaking at 1.5 h, which coincides with the onset of cell division (Fig. 1b). By contrast, the protein levels of ESCRT-III-1 and ESCRT-III-2 reached their maxima ~30 min later (Fig. 1b), consistent with the scenario under which ESCRT-III is the first to arrive to the mid-cell where it recruits ESCRT-III-1 and ESCRT-III-2 (16). Whereas the level of ESCRT-III gradually decreased starting from 1.5 h, those of ESCRT-III-1 and ESCRT-III-2 displayed a 1 h lag phase, maintaining their maximal intensity until 2.5 h time point (Fig. 1b). Importantly, we observed ESCRT-III rings with different diameters. The decrease in diameter coincided with the extent of membrane constriction between the emerging daughter cells, consistent with progression of cytokinesis (Fig. 1c). Notably, ESCRT-III undergoes a relatively slow degradation in *S. islandicus,* contrasting the situation in *S. acidocaldarius*, where rapid degradation of CdvB (ESCRT-III) initiates the membrane constriction by CdvB1 (ESCRT-III-1) and CdvB2 (ESCRT-III-1) (16).

### Dominant-negative mutants of ESCRT-III and Vps4 show division deficiency

To study the roles of ESCRT-III and Vps4 in cell division, we constructed strains expressing dominant-negative mutants of the two proteins from the corresponding plasmids under the control of an arabinose-inducible promoter. Deletion of the ESCRT-III C-terminal winged helix-like domain responsible for the interaction with the C-terminal domain of CdvA (9, 22) produced a mutant, ESCRT-IIIΔC1, incapable of interaction with CdvA (Fig. S1a). We also constructed a dominant-negative mutant of Vps4, Vps4-T148A, in which the conserved threonine residue (T148) of the ATP-binding Walker A motif was replaced with an alanine (Fig. S1b). The recombinant strain thus expresses both the mutant Vps4 and the chromosomally encoded native Vps4. Due to the presence of the residual activity of the wild-type Vps4, the disassembly of the ESCRT-III polymers is slowed down, rather than blocked completely. Therefore, cells at different stages of constriction can be observed.

**Fig 2:**
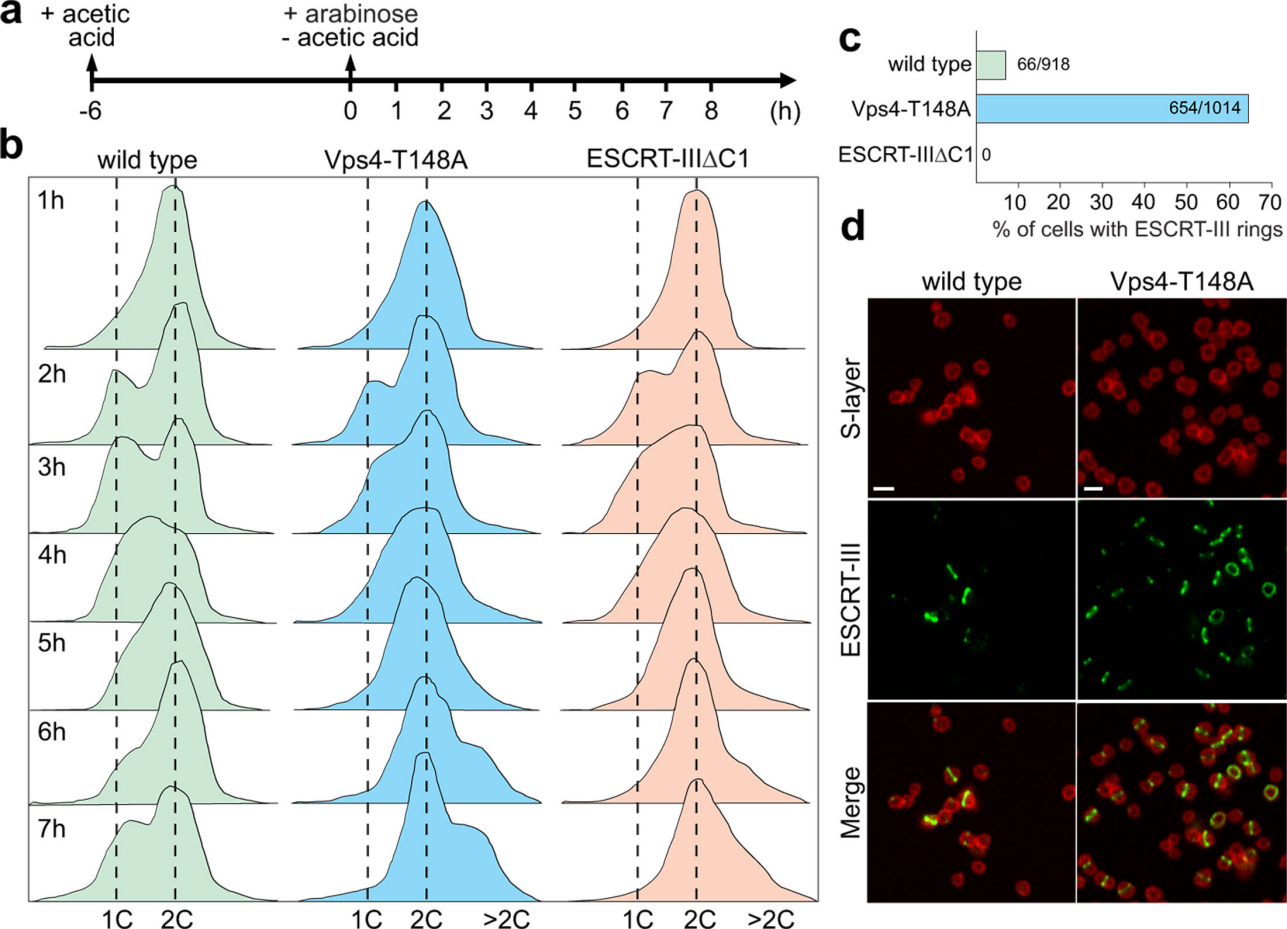
Cells over-expressing the dominant-negative mutants of Vps4 and ESCRT-III display division deficiency. **(a)** Schematic showing the experimental design, with Vps4 and ESCRT-III mutants being over-expressed in synchronized cells. The cells were synchronized to the G2 phase following the treatment with acetic acid for 6 h (−6 to 0 h). Then, acetic acid was removed and arabinose was added at 0 h to induce over-expression of the mutant proteins. **(b)** Flow cytometry analysis of the synchronized cells over-expressing Vps4 and ESCRT-III mutants. Cells harboring the empty vector pSeSD were used as the control. Broken lines denote cells with one and two chromosomal copies, 1C and 2C, respectively. **(c)** Quantification of the ratio of cells with detectable ESCRT-III rings in control cells and cells overexpressing dominant negative Vps4-T148A and ESCRT-IIIΔC1 cells at the 6 h time point. **(d)** Confocal imaging of ESCRT-III in the cells over-expressing the dominant negative Vps4-T148A mutant. Cells harboring the empty vector pSeSD were used as the control. Scale bars, 2 µm.

Expression of the two mutant proteins, Vps4-T148A and ESCRT-IIIΔC1, was induced in cells synchronized at the G2 phase, concomitant with the release of cell cycle arrest (Fig. 2a), with cells harboring the empty vector pSeSD used as a control. Flow cytometry analysis suggested that, under these induction conditions, the first cycle of cell division took place ~2 h following the removal of acetic acid, similar to the control cells, but the second round of division which occurred at ~6 h in the control cells, was blocked in both mutants (Fig. 2b). The differences between the control and mutant cells became obvious after 3 h of induction, suggesting that at this point the pool of mutant proteins reached a concentration sufficient for eliciting a measurable effect on the cell division process (Fig. 2b). Indeed, at 5 h post-induction, nearly all cells contained two copies of the chromosome, whereas at the later time points the population included cells with more than two chromosome copies (Fig. 2b). Prolonged overexpression of the Vps4 mutant had a significant impact on the culture growth (Fig. S2a) and following 12 h or 18 h of overexpression, nearly all cells in the population contained more than two chromosome copies (Fig. S2b). Thus, although the cell cycle could not progress into the cell division phase, genome replication continued in the cells expressing dominant-negative mutants of either ESCRT-III or Vps4. Furthermore, during the advanced stages of cytokinesis, when the cells were connected by a membrane bridge, we observed that DNA was segregated into the emerging daughter cells (see Fig. S5), suggesting that cell division is uncoupled from DNA replication and segregation in *S. islandicus*.

In synchronized cells over-expressing the ESCRT-IIIΔC1 mutant, the formation of the ESCRT-III ring was abolished and the cells grew larger compared to the control cells (Fig. 2c; Fig. S3), suggesting that the interaction between CdvA and ESCRT-III is necessary for the ESCRT-III ring assembly. Furthermore, ESCRT-III-1 and ESCRT-III-2 rings were not observed either (Fig. S3b), indicating that ESCRT-III ring formation is a prerequisite for the recruitment of ESCRT-III-1 and ESCRT-III-2 to the mid-cell, consistent with the interaction between the three ESCRT-III proteins (18).

Upon overexpression of the Vps4-T148A mutant in synchronized cells, the ratio of cells with the ESCRT-III ring-like structures has increased dramatically, from 7.1% (66/918) in the control cells (Sis/pSeSD) to 64.5% (654/1014) in the Vps4-T148A mutant (Fig. 2c and d). These results indicate that Vps4 is necessary for the constriction of the ESCRT-III rings and, accordingly, the cell division progression. In wild-type *Sulfolobus* cells, cytokinesis is a very rapid process, accomplished within ~2 min (17, 29), making it challenging to capture the intermediates at different states of membrane constriction. Thus, the Vps4-T148A mutant strain can serve as a valuable model to study the role of different ESCRT-III homologs during cell division.

### Vps4 disassembles the ESCRT-III ring prior to its degradation by the proteasome

**Fig 3:**
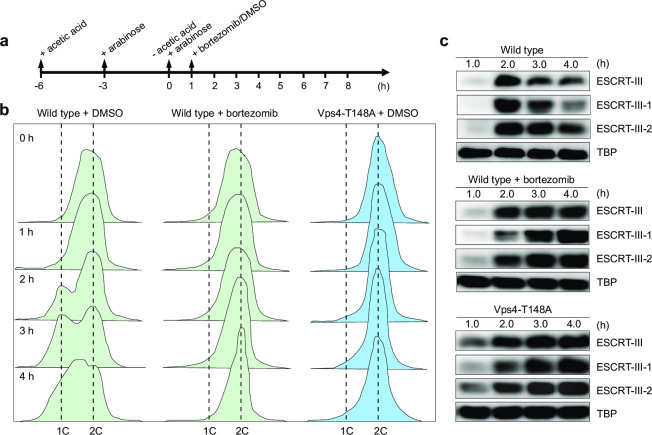
Vps4 disassembles the ESCRT-III filaments, which are then degraded by the proteasome. **(a)** Schematic showing the experimental design. Acetic acid was used to synchronize the cells. Expression of the mutant proteins was induced by the addition of arabinose at −3 h and then re-addition at 0 h. The proteasome inhibitor bortezomib was added at 1 h (0.5 h before cell division) and dimethyl sulfoxide (DMSO) was used as the control. Flow cytometry (b) and western blot (c) analysis of the synchronized cells treated with the proteasome inhibitor and cells over-expressing the dominant-negative Vps4 mutant. Cells harboring the empty vector pSeSD and treated with DMSO were used as a control. Representative images are shown in (c).

To study whether cell division in *S. islandicus* REY15A is triggered by the degradation of ESCRT-III by the proteasome (16), the proteasome inhibitor bortezomib was added to the synchronized cells at 1 h after release of the cell cycle arrest, i.e., 0.5 h before the onset of cell division (Fig. 3a). Cells treated with dimethyl sulfoxide (DMSO) were used as a control. The control cells started to divide ~2 h following the release of the cell cycle arrest (Fig. 3b), with the protein levels of all three ESCRT-III homologs gradually decreasing (Fig. 3c). However, when the proteasome inhibitor was added to the synchronized cells, the division was blocked (Fig. 3b), recapitulating the effect reported for *S. acidocaldarius* (16). The same result was obtained in the absence of the inhibitor in the strain expressing the dominant-negative Vps4 mutant when protein expression was induced 3 h prior to the arrest release (with inducer re-added at time point 0 h). Importantly, protein levels of ESCRT-III, ESCRT-III-1, and ESCRT-III-2 diminished in neither the proteasome inhibitor-treated cells nor the dominant-negative Vps4 mutant (Fig. 3c). These results suggest that Vps4 disassembles the filaments formed by all three ESCRT-III proteins, which is prerequisite for their degradation by the proteasome, which is in line with the results obtained with *S. acidocaldarius* (16).

### ESCRT-III homologs form different assemblies during cell division

**Fig 4:**
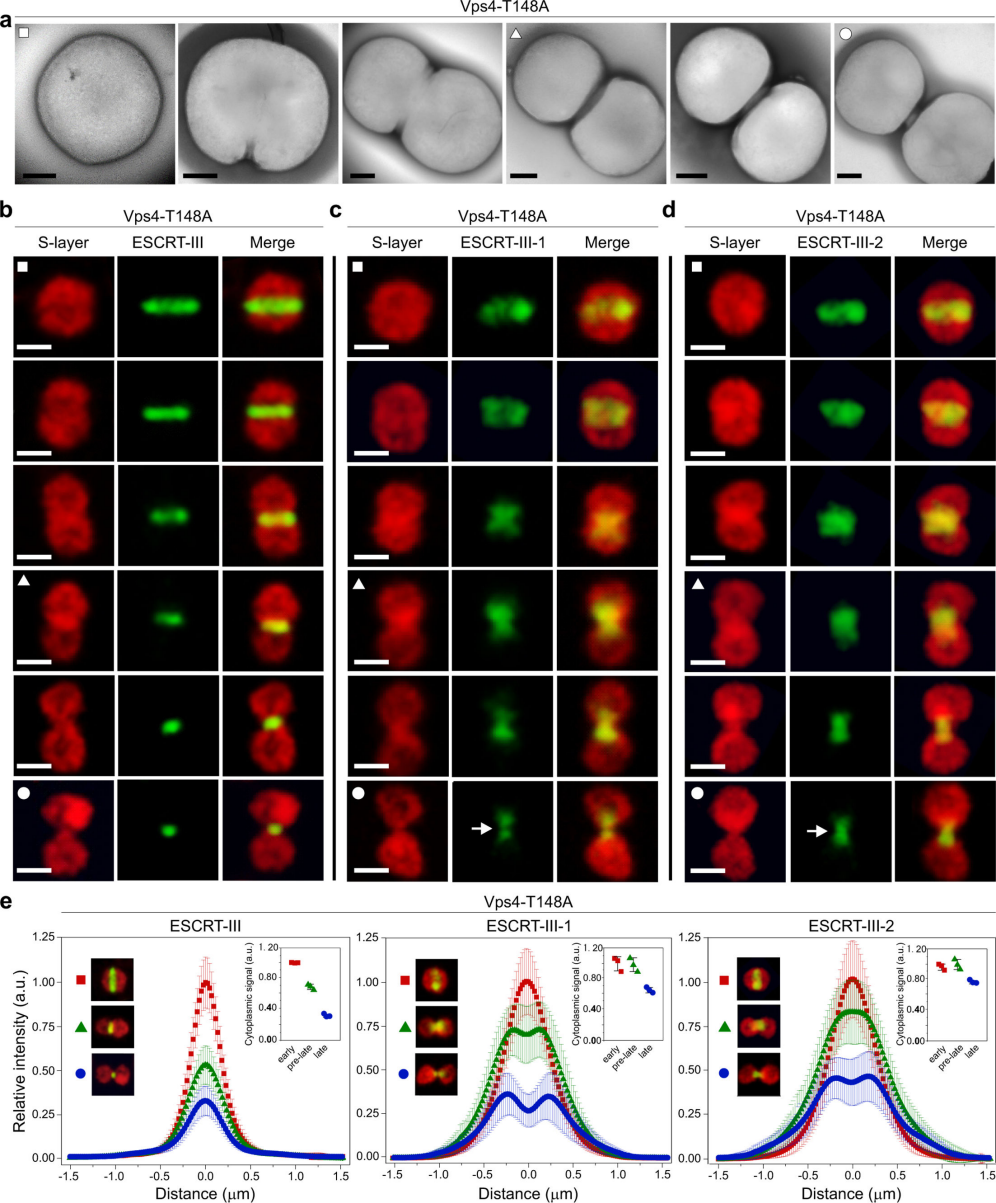
Different stages of cell division visualized in cells overexpressing a dominant negative mutant of the AAA+ ATPase Vps4. **(a)** Representative transmission electron micrographs of cells over-expressing the Vps4-T148A mutant. Scale bars, 200 nm. **(b–d)** *In situ* immunofluorescence confocal microscopy acquisition of a z projection of the ESCRT-III (b), ESCRT-III-1 (c), and ESCRT-III-2 (d) proteins in synchronized cells over-expressing the Vps4 mutant. White arrows in c and d indicate the discontinuity in the ESCRT-III-1 and ESCRT-III-2 fluorescent signals. The cells were collected at 5 h after induction by arabinose as shown in Fig. 2a. Scale bars, 1 µm. “Early,” “pre-late,” and “late” stages of membrane constriction are indicated with white rectangles, triangles, and spheres, respectively. (e) Average intensity profiles of ESCRT-III (left), ESCRT-III-1 (middle), and ESCRT-III-2 (right) rings at different stages of cytokinesis. a.u., arbitrary units. The profiles were obtained from 50 cells using ImageJ. The insets in each panel show the cytoplasmic signal of the corresponding ESCRT-III homologs at different stages of cytokinesis. The measurements were done in triplicate with each replicate based on 16 cells. Symbol designations as in panels b–d.

Examination of cells expressing Vps4-T148A by electron microscopy revealed that the population contains cells at different stages of cytokinesis (Fig. 4a). This was confirmed by confocal microscopy analysis which showed that ESCRT-III formed rings with different diameters corresponding to different extents of membrane constriction (Fig. 4b), closely resembling those observed in the wild-type cells (Fig. 1c). Similarly, ESCRT-III-1 and ESCRT-III-2 also formed rings with different diameters in the Vps4-T148A mutant (Fig. 4c and d). Notably, in the confocal microscopy analysis, the rings formed by ESCRT-III-1 and ESCRT-III-2 had a different appearance compared to those of ESCRT-III and likely underwent rearrangements as membrane constriction progressed. During the initial stages of constriction, ESCRT-III-1 and ESCRT-III-2 each formed bands, roughly twice wider than the discrete ESCRT-III ring (Fig. 4e; Fig. S4). Subsequently, these wide bands transformed into assemblies resembling inverted cones extending away from the central constriction site, with two fluorescence intensity peaks flanking the central area occupied by ESCRT-III, suggesting that ESCRT-III-1 and ESCRT-III-2 formed double rings or spirals (Fig. 4b through e). Notably, in the cells that reached an advanced stage of cytokinesis (i.e., rings with a small diameter), the fluorescence signal for ESCRT-III-1 and, to a lesser extent, ESCRT-III-2 was not continuous, suggesting that the two proteins form assemblies on either side of the central division furrow, likely occupied by ESCRT-III. In *S. acidocaldarius*, the clearance of the ESCRT-III ring is a prerequisite for membrane constriction to occur (24). However, in *S. islandicus* REY15A, the degradation of ESCRT-III appears to be gradual and concomitant with constriction. In particular, there is about 70% and 30% of the ESCRT-III content present in cells at the pre-late and late stages of constriction, respectively (Fig. 4e, insets). Notably, there is no obvious degradation of ESCRT-III-1 and ESCRT-III-2 until the cells enter into the late stage of division (Fig. 4e). These results suggest different roles during cell division for ESCRT-III on one hand and the other two homologs on the other and further reinforce the notion that ESCRT-III does not only serve as a platform for recruitment of ESCRT-III-1 and ESCRT-III-2 but also plays an active role during membrane constriction.

Notably, upon prolonged (12 h) overexpression of the dominant negative Vps4 mutant, cells became considerably larger in size (average diameter of 2.73 µm; *n* = 307 cells) compared to the control cells (~1.2 µm) (25) (Fig. S3a). Interestingly, multiple ESCRT-III rings were observed in these cells. Typically, the central ring had a smaller diameter and was surrounded by two larger, similarly spaced rings on either side (Fig. S5a). By contrast, the fluorescent signal corresponding to ESCRT-III-2 appeared as a band extending across the entire area of the division furrow (Fig. S5b). Like in the case of ESCRT-III rings (single vs multiple), this phenotype differs from that observed in normal-sized cells in which the Vps4 mutant was expressed for 5 h (Fig. 4d), instead of 12 h (Fig. S5b). The differences in the ESCRT-III-2 appearance under the two conditions may be linked to differences in the dimension of the division furrow, which appears to be longer in the enlarged cells (Fig. S5b). Collectively, these results emphasize the different organizations of the filaments formed by the ESCRT-III paralogs during cell division. Thus, whereas overexpression of the dominant negative mutant of ESCRT-III abolished cell division at the earliest stage, the Vps4-T148A mutant interfered with the disassembly of the ESCRT-III ring, producing a population of cells blocked at different stages of cell division.

### ESCRT-III-1 and ESCRT-III-2 are necessary for the final abscission

**Fig 5:**
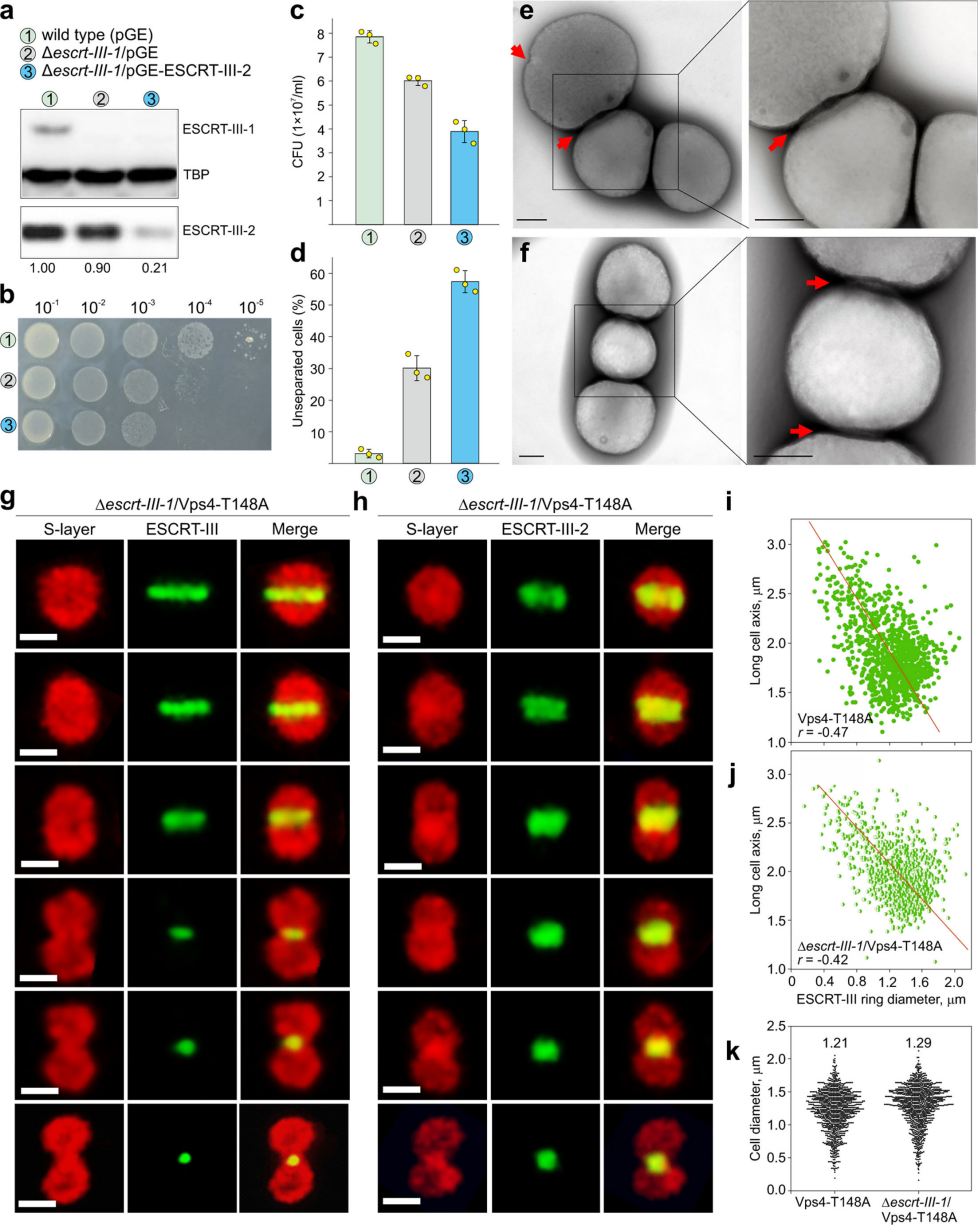
ESCRT-III-1 is dispensable for the initial stages of membrane constriction but becomes important at the later stage of membrane abscission. The three strains compared throughout this figure are denoted as 1, 2, and 3, and correspond to (**i**) the wild-type control strain carrying the empty vector pGE, (ii) the *escrt-III-1* deletion strain carrying the empty vector pGE (Δescrt-III-1/pGE), and (iii) the *escrt-III-1* deletion strain in which the expression of ESCRT-III-2 has been knocked down using the endogenous type III CRISPR system and specific CRISPR spacers expressed from the plasmid pGE-ESCRT-III-2 (Δescrt-III-1/pGE-ESCRT-III-2). **(a)** Western blot analysis of ESCRT-III-1 and ESCRT-III-2 in the three different strains. TBP was used as the loading control. The numbers under the western blot indicate the expression levels of ESCRT-III-2 in the three strains. **(b)** Spot test showing the growth of the three strains. **(c)** Colony formation by the three strains. Error bars represent the standard deviation from three independent experiments. One thousand cells were plated on the solid MATV [mineral salts (M) with 0.2% (wt/vol) arabinose (A), 0.2% (wt/vol) tryptone (T), and a mixed vitamin solution (V)] medium and the colonies were counted after 7 days of cultivation. **(d)** The ratio of unseparated cells blocked during different stages of cell division. More than 300 cells were counted. Error bars represent the standard deviation from three independent experiments. **(e–f)** Representative transmission electron micrographs of Δ*escrt-III-1* cells (e) and cells over-expressing the dominant-negative mutant of ESCRT-III-1 in which the C-terminal MIMI2 domain has been deleted (f). Red arrows point to the sites of membrane constriction. Scale bars, 500 nm. **(g–h)** *In situ* immunofluorescence confocal microscopy acquisition of a z projection of ESCRT-III (g) and ESCRT-III-2 (h) rings in the *escrt-III-1* deletion mutant cells over-expressing the dominant-negative Vps4-T148A mutant. Immunofluorescent labeling of the S-layer, ESCRT-III and ESCRT-III-2 is shown in different panels along with the merged images. Scale bars, 1 µm. (**i–j)** Correlation between the ESCRT-III ring diameter and the long axis of the cells in the wild type and Δescrt-III-1 cells overexpressing the dominant negative Vps4-T148A mutant. One thousand cells were counted in each case. The best-fit line is shown in red. *r,* Pearson correlation coefficient. **(k)** Violin plots show the distribution of the cell diameters in the wild type and Δ*escrt-III-1* cells overexpressing the dominant negative Vps4-T148A mutant. The numbers above the graphs indicate the average diameters.

Given that ESCRT-III appears to play an active role in membrane constriction, we set out to investigate the contribution of ESCRT-III-1 and ESCRT-III-2 in this process. To this end, we first constructed the *escrt-III-1* deletion mutant using the CRISPR-based method. The Δ*escrt-III-1* cells formed fewer colonies on a solid medium and there was an increased fraction of unseparated cells compared to the wild type (Fig. 5a through d), suggesting defects at the late stages of cytokinesis. Observation of the Δ*escrt-III-1* cells under an electron microscope indeed revealed “chains” of unseparated cells, with some of the cells undergoing the next round of cytokinesis prior to the completion of the first one (Fig. 5e). A similar phenotype was reported by us previously with the partial deletion mutant of *escrt-III-1* (18). A “chain-like” phenotype was also obtained upon expression of a dominant-negative mutant of ESCRT-III-1 in which the C-terminal MIM2 domain (responsible for interaction with the MIT domain of Vps4) was deleted (Fig. 5f). These results suggest that ESCRT-III-1 plays an important role that affects the pre-late stage of cytokinesis when the cell is constricted to approximately 1/3 of its initial diameter, but, unlike ESCRT-III and ESCRT-III-2, this protein is dispensable for the initiation of membrane constriction.

To analyze whether *escrt-III-1* deletion affects the localization and ring formation of ESCRT-III and ESCRT-III-2, we over-expressed the dominant-negative mutant of Vps4 (to increase the ratio of cells with observable rings) in the Δ*escrt-III-1* cells. As shown in Fig. 5g and h, ESCRT-III and ESCRT-III-2 formed rings of different diameters at the mid-cell in the absence of ESCRT-III-1, indistinguishable from those obtained in the wild-type ESCRT-III-1 background (Fig. 3). Whereas, the ESCRT-III ring appeared as a distinct band occupying a central position at the constriction site, ESCRT-III-2 formed a wider, presumably double ring on both sides of the constriction site. The diameters of ESCRT-III rings inversely correlated with the length of the cell axis in the presence and absence of ESCRT-III-1, signifying similar constriction at the mid-cell (Fig. 5i through k). These results suggest that ESCRT-III-1 plays little if any role in the positioning and formation of the ESCRT-III and ESCRT-III-2 rings as well as initiation of membrane constriction in *S. islandicus* cells. However, we cannot exclude the possibility that it facilitates this process.

**Fig 6:**
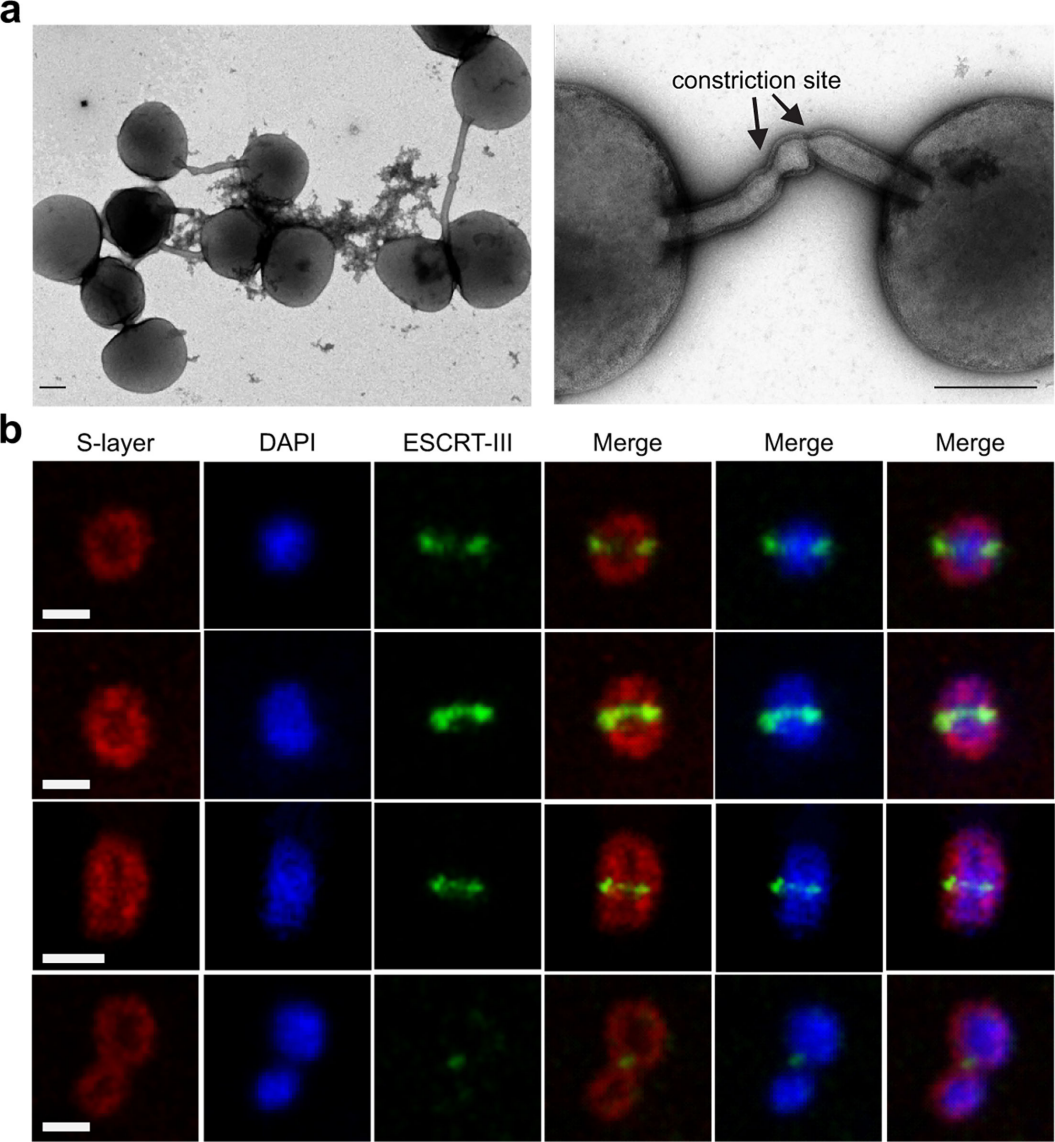
ESCRT-III-2 is dispensable at the initial stage of membrane constriction, but essential for the final membrane abscission. **(a)** Representative transmission electron micrographs of cells over-expressing the dominant-negative mutant of ESCRT-III-2 in which the C-terminal MIMI2 domain has been deleted. Arrows point to the sites of membrane abscission. Scale bars, 500 nm.** (b)** *In situ* immunofluorescence confocal microscopy acquisition of a z projection of *escrt-III-1* deletion strain in which the expression of ESCRT-III-2 has been knocked down using the endogenous type III CRISPR system and specific CRISPR spacers expressed from the plasmid pGE-ESCRT-III-2 (Δescrt-III-1/pGE-ESCRT-III-2). Scale bars, 2 µm.

We next assessed the role of ESCRT-III-2 in membrane constriction. The gene encoding ESCRT-III-2 is indispensable in *S. islandicus* REY15A (18, 30). Thus, we expressed a dominant-negative mutant of ESCRT-III-2 in which the C-terminal MIM2 domain was deleted. Electron microscopy analysis revealed cells arrested at the late stage of cytokinesis, in which the daughter cells were connected through membrane bridge-like structures (Fig. 6a). Interestingly, the latter often contained structures superficially resembling the midbody-like structures typical of eukaryotic cytokinesis. Notably, in Vps4-T148A overexpressing cells, ESCRT-III-2 is distributed along the length of the narrow tubes connecting the daughter cells (Fig. S5b). Finally, to study whether membrane constriction can proceed in the absence of both ESCRT-III-1 and ESCRT-III-2, we constructed an arabinose-inducible ESCRT-III-2 knockdown strain in the Δ*escrt-III-1* background. The expression level of ESCRT-III-2 was depleted to 21% of the control level after induction with arabinose for 12 h (Fig. 5a). Cells with both *escrt-III-1* deletion and ESCRT-III-2 knockdown formed even fewer colonies on the solid medium than the Δ*escrt-III-1* cells (Fig. 5b and c), and the fraction of unseparated cells further increased (Fig. 5d). Nevertheless, immunofluorescence microscopy analysis suggested that ESCRT-III could still form contractile rings with variable diameters (Fig. 6b). Based on the diameter of the membrane connection between the dividing daughter cells in Δ*escrt-III-1* and ESCRT-III-2 knockdown strains, it appears that ESCRT-III-1 acts upstream of ESCRT-III-2. Collectively, these results suggest that ESCRT-III-1 and ESCRT-III-2 are important for the final stages of cytokinesis, facilitating progression from the “pre-late” to “late” and from the “late” to final abscission, respectively. However, we cannot exclude the possibility that ESCRT-III plays an auxiliary role during the final abscission, which would be consistent with the low ESCRT-III signal in cells that fail in abscission (Fig. 6b).

### Super-resolution microscopy shows that ESCRT-III rings do not spiral upon constriction

The ESCRT-III rings have apparent thicknesses of only 400 nm (Fig. S4), i.e., close to the resolution of confocal microscopes. To study whether ESCRT-III rings undergo reorganization during constriction, we therefore performed 3D SMLM, using a custom-built microscope and the ZOLA-3D reconstruction software (31) (see Materials and Methods and Fig. S6). To quantitatively characterize the ring-like structures observed by 3D SMLM, we developed a dedicated analysis procedure that measures the ring circumference and thickness from the localization data while allowing for deviations from circularity or planarity (see Materials and Methods and Fig. S7). Simulations of ring-like structures of various sizes and orientations that account for the estimated localization precision of our data suggest that we can resolve thicknesses above 40 nm (see Materials and Methods and Fig. S7).

**Fig 7:**
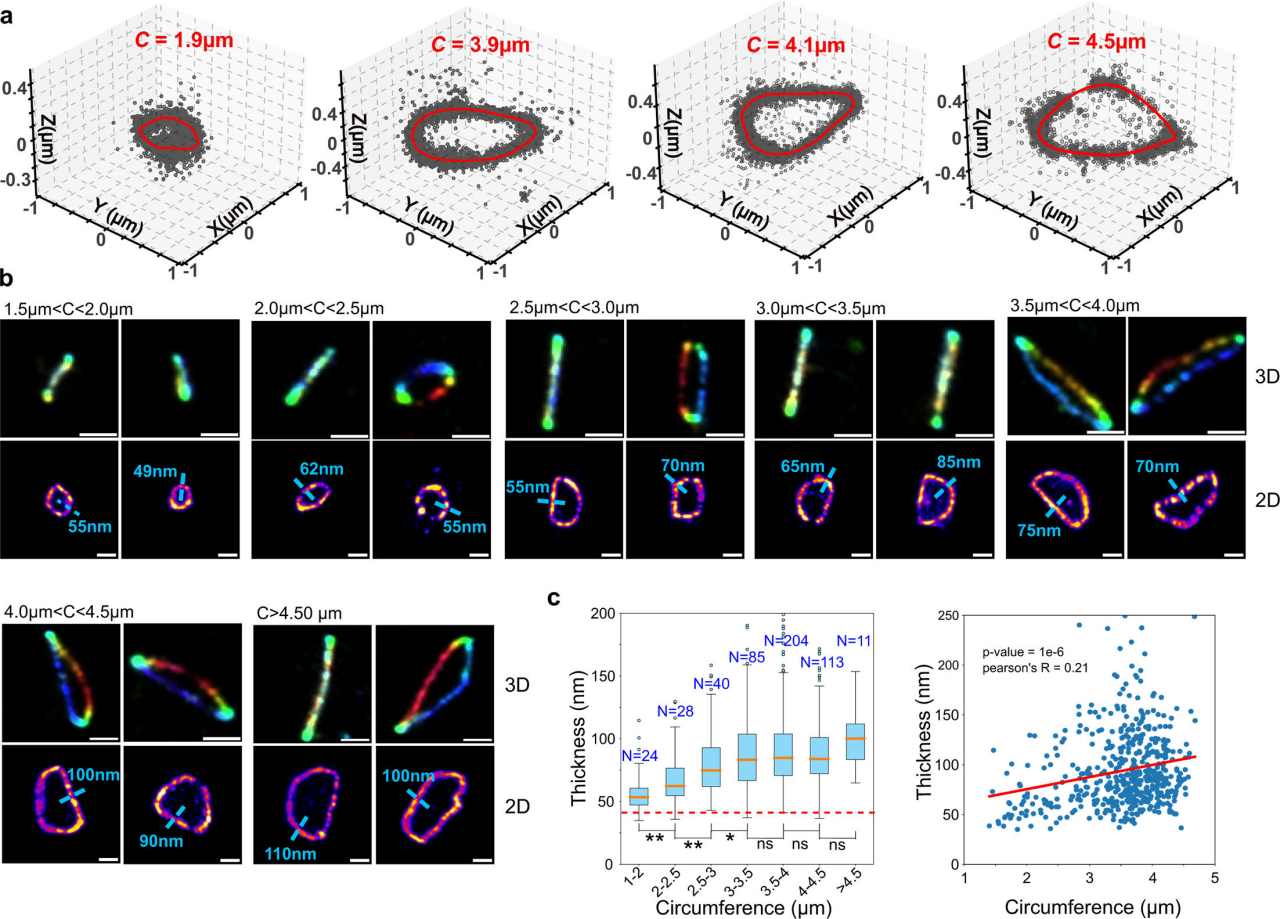
Super-resolution characterization of ESCRT-III rings in the cells overexpressing the Vps4-T148A mutant. (**a)** 3D scatter plots (black dots) show SMLM localizations of ESCRT-III rings for four distinct cells of different sizes. For each cell, the set of 3D localizations was rotated to bring them in close proximity to the XY plane, and then a closed curve (red) was fitted to the localizations (see Materials and Methods). The circumference (***C***) of each fitted curve is indicated. **(b)** Color-coded 3D views (top rows) and rotated 2D views of ESCRT-III rings (bottom rows) are shown. Scale bars, 250 nm. The thickness of the ring was measured at manually selected locations (dashed blue lines) as indicated. Additional examples can be found in Fig. S8b. (c) Box plots show the distribution of the calculated ESCRT-III ring thickness for different ranges of calculated ring circumferences. The dashed red line shows the minimal thickness (resolution limit) estimated from simulated data (see Fig. S7d) using the same algorithm as for the experimental data, and assuming an antibody labeling size of 17.5 nm and a localization precision of 25 nm. The orange lines in the box plots indicate the mean values. The number of cells (**N**) for each range of circumferences is indicated. A total of 513 cells were analyzed. Statistical significance was assessed using the Wilcoxon test (*, *P* < 0.05; **, *P* < 0.01; ns, not significant; *P* > 0.05). The scatter plot shows the calculated ring thickness vs the calculated circumference (each dot corresponds to a single cell). The red line shows a linear fit, along with a *P*-value indicating significant correlations.

To increase the ratio of cells with ESCRT-III rings, we analyzed cells expressing the dominant-negative Vps4-T148A mutant, induced for 5 h (Fig. S8a). Although ESCRT-III rings exhibited punctated fluorescence, the labeling allowed unequivocal tracing of their contours. A total of *n* = 513 ESCRT-III rings from 16 fields of view in two independent experiments were randomly selected and manually cropped for quantitative analysis (Fig. 7a through C; Fig. S8b). Fig. 7b shows ESCRT-III rings grouped according to their circumference (see Fig. S8b for additional examples), which ranged from 1.5 to 2.0 μm (corresponding to diameters of ~0.4–0.6 µm) to >4.5 µm (diameter >1.4 µm). These values are within the range observed for the wild-type *S. islandicus* REY15A cells, where most non-dividing cells are ~1.2 µm in diameter (25). Under these expression conditions, a single ESCRT-III ring was observed in all cells (Fig. S8a).

To analyze whether membrane constriction is accompanied by a rearrangement of the ESCRT-III ring, we analyzed the thickness of the ESCRT-III rings as a function of the ring circumference (*C*). No significant changes in the ring thickness were observed for rings with circumferences ranging between 3.0 µm and 4.5 µm (Fig. 7c). However, smaller rings (*C* < 3.0 µm) were significantly thinner than the larger rings: we observed a gradual and significant decrease in thickness from ~83 nm for the *C* = 3.0–4.5 µm rings to ~74 nm for the *C* = 2.5–3.0 µm rings (Wilcoxon-test *P* < 0.05) and ~52–61 nm for the *C* < 2.5 µm rings (*P* < 0.01) (Fig. 7c). These differences in automatically measured ring thickness were also confirmed by manual measurements of the ring thickness based on line profiles (Fig. 7b; Fig. S8b). This data suggests that before a ring circumference of ~3 µm is reached, the ESCRT-III ring contraction is not accompanied by major structural reorganization, at least at the resolution of our imaging technique. However, after reaching this threshold, further constriction involves a significant thinning of the ESCRT-III ring.

It has been suggested that membrane deformation and fission are driven by polymerization of eukaryotic ESCRT-III homologs into multistranded spring-like spirals (32). If this was the case for *S. islandicus*, then depending on whether neighboring strands are resolvable by SMLM or not, we would expect rings of smaller size to either exhibit spiral-like structures or larger thicknesses, respectively. Since we observe the opposite, namely that smaller rings are thinner, we can exclude the reorganization of the ESCRT-III ring into a multistranded spiral. Instead, our data are consistent with a progressive depolymerization of the ESCRT-III filament upon constriction.

As mentioned above (Fig. S5), cells overexpressing the dominant-negative Vps4-T148A mutant for 12 h displayed multiple ESCRT-III rings. Thus, we leveraged the superior resolution of 3D SMLM to verify whether these multiple rings are interconnected, i.e., represent a spiral, or are discrete. We imaged cells with up to four rings (Fig. S9**,** Supplementary data file 1), whose circumference varied from 3 to 6 µm (corresponding to major axis diameters of 1 to 2 µm). Notably, the mean thickness of the rings is 95 nm as evaluated from 20 cells. This thickness is similar to that in cells containing a single ring. Furthermore, although our resolution would be sufficient to see connections between the rings, no such connections were observed. These results suggest that excess ESCRT-III (in the absence of its proteasomal degradation) leads to the formation of additional rings rather than the formation of a spiral or thickening of the ring.

## DISCUSSION

**Fig 8:**
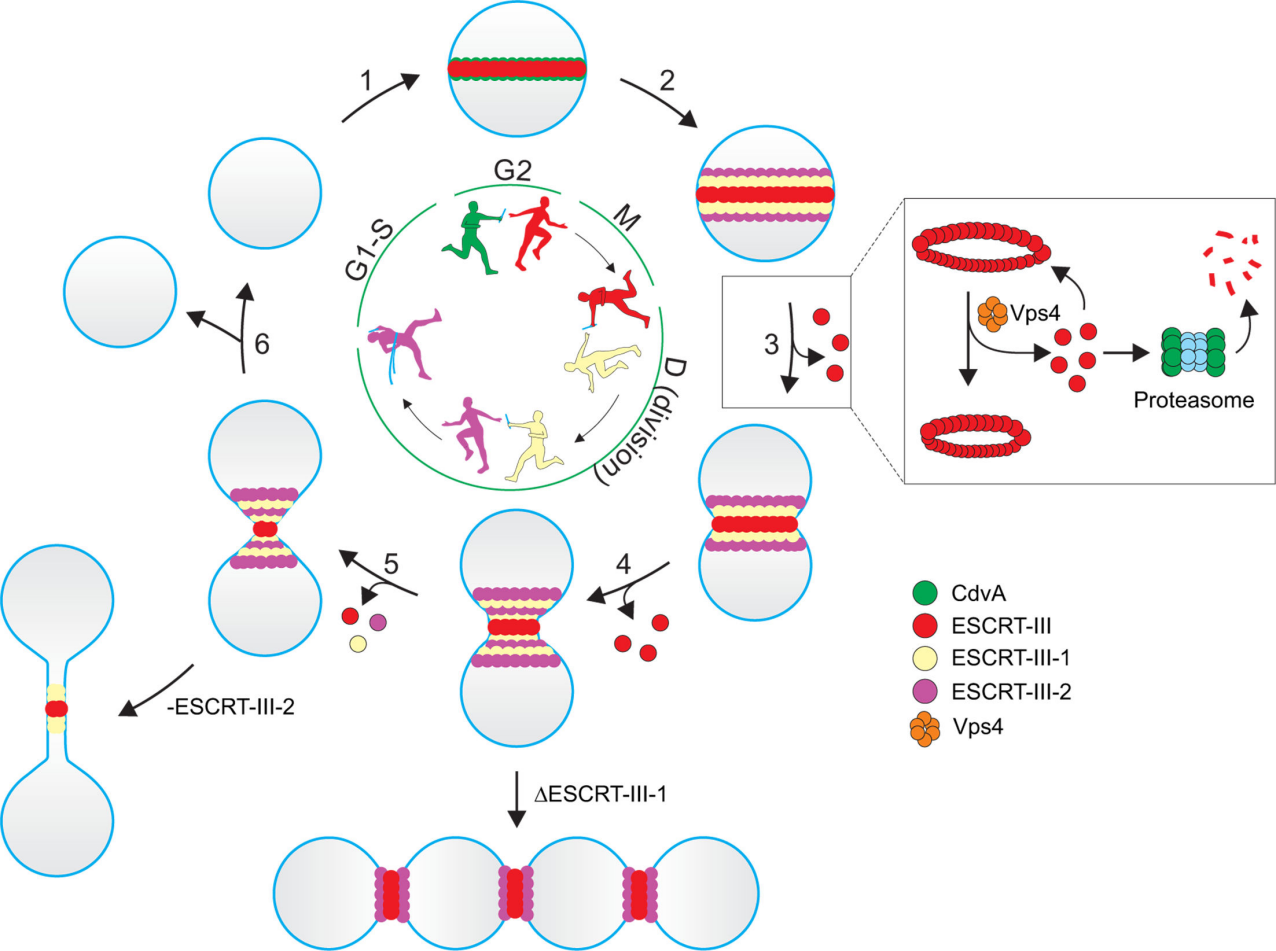
Relay race model of cell division in *S. islandicus*. Different stages of cell division are numbered and the major participants of the relay race are color-coded, with the legend provided on the right side of the figure. Silhouettes in the middle represent cell division proteins that are colored with the corresponding colors, whereas the membrane (cyan) is represented by a baton which is passed during the relay race. (1) The process starts with the recruitment of ESCRT-III (red) to the mid-cell by CdvA (green). (2) ESCRT-III in turn interacts with and recruits ESCRT-III-1 (yellow) and ESCRT-III-2 (magenta), which form rings on both sides of the central ESCRT-III ring. (3) Ring constriction commences when the Vps4 ATPase (orange) initiates disassembly of the ESCRT-III ring. Inset: If disassembled ESCRT-III subunits are not degraded by the proteasome, they repolymerize into the ESCRT-III and the Vps4 enters into a futile cycle, and cell division is blocked. (4) Once a cell is constricted to approximately 1/3 of its initial diameter, the constriction is passed on to ESCRT-III-1. Further constriction is accompanied by reorganization of the ESCRT-III-1 and ESCRT-III-2 rings, presumably into spirals, whereas ESCRT-III only decreases in diameter. (5) Disassembly and degradation of all three ESCRT-III homologs leads to further constriction. Once a narrow membrane neck is achieved, membrane scission is performed by ESCRT-III-2; in the absence of ESCRT-III-2, the cells remain connected through narrow-diameter membrane bridges. (6) Membrane is abscised and two daughter cells are produced.

Based on our results, we propose the “relay race” model of *S. islandicus* cell division (Fig. 8). The process starts with CdvA interaction with ESCRT-III and the formation of a ring-like structure at the mid-cell. Indeed, deletion of the ESCRT-III C-terminal winged helix-like domain responsible for the interaction with the C-terminal domain of CdvA (9, 22) abolished the interaction with CdvA and aborted the formation of the ESCRT-III ring. In the absence of the ESCRT-III ring, the localization of ESCRT-III-1 and ESCRT-III-2 to the mid-cell was also compromised. This part of the pathway is similar to the *S. acidocaldarius* system. However, unlike in *S. acidocaldarius*, where ESCRT-III-1 and ESCRT-III-2 rings constrict only following the degradation of the static ESCRT-III ring (16), in *S. islandicus*, ESCRT-III appears to play a more active role during the initial stages of membrane constriction. Another apparent difference between the divisions of *S. islandicus* and *S. acidocaldarius* is the architecture of ESCRT-III rings. In *S. islandicus*, the ESCRT-III ring occupies the central position at the constriction site, with the ESCRT-III-1 and ESCRT-III-2 rings being assembled on both sides of the ESCRT-III ring. By contrast, in *S. acidocaldarius*, CdvB flanks the constriction site at the periphery, CdvB2 preferentially localizes to mid zone, and CdvB1 spans the entire length of the bridge (24). This different positioning of the ESCRT-III ring likely underlies the functional differences in the two genera. Indeed, *Sulfolobus* and *Saccharolobus* species display different phenotypes when orthologous components of the ESCRT machinery are deleted, suggesting the existence of mechanistic differences between these relatively closely related organisms. For instance, whereas deletion of the *escrt-III-3* gene had a profound effect on cell division in *S. acidocaldarius* (33), its deletion in *S. islandicus* had no effect on cell growth or division (18, 26, 30). Conversely, whereas *escrt-III-2* could be deleted in *S. acidocaldarius* (33), albeit with observable effects on cell division (17), the gene is indispensable in *S. islandicus* (18, 30).

We were able to capture different intermediates of membrane constriction, where ESCRT-III was consistently localized at the constriction sites. Importantly, deletion of the *escrt-III-1* and ~80% depletion of ESCRT-III-2 did not preclude the initiation of membrane constriction, although we cannot exclude the possibility that the kinetics or efficiency of this process was affected. Regardless, the mutant cells displayed defects during the later stages of cytokinesis. In the *escrt-III-1* deletion mutant, the cell division was blocked when the constriction reached ~30% of the initial cell diameter, a stage we refer to as “pre-late.” By contrast, in cells overexpressing a dominant-negative variant of the ESCRT-III-2 protein, the division was blocked at the final stage of membrane abscission, with daughter cells being connected through narrow membrane bridges. Furthermore, cells depleted for both ESCRT-III-1 and ESCRT-III-2 exhibited a phenotype similar to that of the *escrt-III-1* deletion mutant, suggesting that ESCRT-III-1 functions upstream of ESCRT-III-2. Different eukaryotic ESCRT-III polymers, depending on subunit composition, produce a variety of filament geometries with an inherent propensity to interact with different membrane topologies, template dimensions, and membrane compositions (34). In a similar fashion, archaeal ESCRT-III-1 and ESCRT-III-2 proteins are likely to display different preferences for membrane curvature compared to ESCRT-III. This is consistent with our previous results suggesting that the budding of extracellular membrane vesicles in *S. islandicus* primarily depends on ESCRT-III-1 and ESCRT-III-2 rather than ESCRT-III (26). Thus, under the “relay race” model, the cytokinesis is achieved through a sequential and concerted action of different ESCRT machinery components. In particular, CdvA brings ESCRT-III to the mid-cell, where the latter is important for the initial membrane contraction until a threshold curvature is reached, at which point this role is passed on to ESCRT-III-1 and eventually to ESCRT-III-2, which performs the final membrane abscission, resulting in cell division.

Similar to *S. acidocaldarius*, degradation of ESCRT-III is a prerequisite for cell division. However, our results suggest that besides ESCRT-III, ESCRT-III-1, and ESCRT-III-2 also undergo the same fate. Quantification of the three ESCRT-III homologs during the cell cycle showed that while ESCRT-III is gradually degraded from the onset of cell division, degradation of ESCRT-III-1 and ESCRT-III-2 has a lag phase. We hypothesize that the stable ESCRT-III-1 and ESCRT-III-2 content signifies the rearrangement of these rings into spiral-like structures which appear as inverted cones in confocal microscopy. Following the lag phase, ESCRT-III-1 and ESCRT-III-2 undergo Vps4-mediated disassembly and proteasomal degradation, which may coincide with pre-late and late phases of membrane constriction. Indeed, inhibition of the proteasome with bortezomib led to the accumulation of all three ESCRT-III homologs, as evidenced by western blotting. This is in agreement with the recently reported results with *S. acidocaldarius*, where ESCRT-III-1 and ESCRT-III-2 are degraded in a proteasome-dependent manner during G1 (16). Vps4 appears to play a key role in this process and acts upstream of the proteasomal degradation of the ESCRT-III subunits. Indeed, inhibition of the proteasome and overexpression of the dominant-negative mutant of Vps4 yield the same phenotypes. Given that bortezomib does not affect the Vps4 activity directly, we conclude that once removed from the polymeric ring by Vps4, ESCRT-III subunits re-polymerize into the ESCRT-III ring, and hence the Vps4-mediated ESCRT-III remodeling succumbs to a futile cycle, whereby removed ESCRT-III subunits repolymerize (Fig. 8). Prolonged overexpression of the dominant-negative Vps4 mutant results in accumulation of enlarged cells with increased chromosomal content (>2 copies) and the same phenotype was obtained with the dominant-negative ESCRT-III mutant, suggesting that cell division and DNA replication are not tightly coupled in *S. islandicus*.

Notably, in the enlarged cells overexpressing the dominant-negative Vps4 mutant for extended periods (~12 h), more than one ESCRT-III ring was observed, with the individual rings being disconnected from each other (Fig. S5 and S8). Typically, the ESCRT-III ring was located in the center of the cleavage furrow. Under no conditions tested could we observe a spiral of ESCRT-III. The 28 nm resolution achieved using the 3D SMLM technique applied in this study does not allow us to determine the exact molecular architecture of the ESCRT-III rings, but it is sufficient to rule out the possibility of the spiral formation, i.e., coiling of the initial polymer as the cell constricts, because such a coil would be expected to become thicker. Indeed, smaller rings were nearly twice thinner than the large ones. Interestingly, the initial decrease in ring circumference (C > 3.0–4.5 µm) was not accompanied by changes in the ring thickness, but once the circumference reached less than 3 µm, there was a significant decrease in the ESCRT-III ring thickness. Conceivably, the changes in the ESCRT-III ring thickness are linked to the relegation of the membrane constriction to ESCRT-III-1 and eventually to ESCRT-III-2. Unfortunately, we could not establish conditions for high-quality 3D SMLM imaging of ESCRT-III-1 and ESCRT-III-2 rings, thus this hypothesis requires further investigation.

Recent structural studies on the eukaryotic ESCRT-III proteins CHMP2A and CHMP3 showed that the N-terminal regions of the CHMP2A–CHMP3 subunits in the heteropolymeric helical filament are inserted into the membrane, whereas the C-termini face the lumen of the tube, where they are accessible to Vps4 (35). It was suggested that remodeling of the CHMP2A–CHMP3 polymer by Vps4 induces filament sliding facilitated by electrostatic interfilament interactions, driving membrane constriction (35). The constriction of the FtsZ rings during bacterial cell division also involves sliding of short FtsZ filaments constituting the FtsZ ring past each other through polarized plus-end polymerization and minus-end depolymerization, with GTP hydrolysis setting the rate of depolymerization (36–38). Notably, similar to *S. islandicus* ESCRT-III rings with 4.5 ≥ *C* > 3.0, constriction of the FtsZ ring does not involve a decrease in the ring thickness (39). We hypothesize that a similar mechanism might underlie the initial membrane constriction by ESCRT-III, with the ESCRT-III ring being composed of overlapping filaments, which slide past each other as a result of depolymerization mediated by the Vps4 ATPase. A theoretical model has been proposed whereby membrane constriction is driven by the perversions and supercoiling of ESCRT-III-1 and ESCRT-III-2 filaments (40). At least in the case of ESCRT-III ring, which appears to remain throughout the membrane constriction process, we did not observe local increases in ring thickness, as would be expected in the regions of supercoiling or perversions. Instead, at the smaller ring diameters, we observed that the ESCRT-III rings became twice thinner compared to the larger rings. By contrast, as the cell diameter decreases, ESCRT-III-1 and ESCRT-III-2 rings undergo rearrangement into cones, which likely represent spirals of the corresponding filaments.

Collectively, our results clarify the role of different ESCRT machinery components during the cell division of *S. islandicus* and highlight certain differences with the cytokinesis process reported in *S. acidocaldarius*. Our data suggests that, unlike in the latter model, ESCRT-III plays a more active role during the initiation of membrane constriction, whereas ESCRT-III-1 and ESCRT-III-2 appear to primarily play more specialized roles during the later stages of membrane abscission, although the auxiliary role of ESCRT-III at the final abscission cannot be ruled out. The differences between the two species emphasize the plasticity of the archaeal ESCRT machinery. Further research on both systems is likely to explain the intricate mechanistic details underlying these differences.

## MATERIALS AND METHODS

### Strains, growth conditions, and transformation of *Saccharolobus*

*Saccharolobus islandicus* REY15A was grown aerobically with shaking (145 rpm) at 75°C in STV medium containing mineral salts (M), 0.2% (wt/vol) sucrose (S), 0.2% (wt/vol) tryptone (T), a mixed vitamin solution (V); E233S (REY15A*ΔpyrEFΔlacS*) was grown in STVU medium containing additional 0.01% (wt/vol) uracil (U); the pH was adjusted to 3.5 with sulfuric acid, as described previously (26). SCV medium containing 0.2% (wt/vol) casamino acids (C) was used for the selection of uracil prototrophic transformants. ATV medium containing 0.2% (wt/vol) arabinose (A) was used to induce protein overexpression. The plasmids and strains constructed and used in this study are listed in Tables S1 and S2, respectively.

For the spot test, cell cultures of Sis/pSeSD and *Δescrt-III-1*/pSeSD were cultivated to an OD_600_ of around 0.2. Then 10 µL of the 10 times gradient diluted cell cultures were spotted on the pre-warmed MSCV plates and incubated at 75°C for 3–5 days.

### Construction of the CRISPR-based gene knockout plasmids and gene knockout

The CRISPR type I-A-based gene knockout plasmids (Table S1) were constructed according to the methods described previously (41, 42). For gene knockout, 40-nt protospacers matching the genes of interest were selected from the sense or anti-sense strand of the corresponding genes downstream of the CCN or TCN (5′−3′) sequences and cloned into the genome-editing plasmid pGE (41). The spacers selected and used in this study are listed in Table S3. The L-arm and R-arm of the targeted genes were inserted into the plasmid after spacer insertion and the primers used to amplify the L-arm and R-arm are listed in Table S3. The knockout plasmids were introduced into E233S cells by electroporation and transformants were selected on MSCV plates without uracil.

### Construction of site-directed ATP binding mutant of Vps4

The fragment of the ATP binding mutant of Vps4 (Vps4-T148A) was amplified by overlap extension PCR, with the restriction sites of NdeI and SalI located at the 5′- and 3′-terminal, respectively. After double enzyme digestion by NdeI and SalI (Thermo Fisher Scientific, USA) at 37°C overnight, the fragment was inserted into the over-expression plasmid pSeSD by the T4 DNA ligase (Promega, USA). After sequencing, the plasmid pSeSD-Vps4-T148A was transferred into E233S by electroporation and the transformants were selected on MSCV plates without uracil.

### Cell synchronization and cell cycle analysis

*S. islandicus* REY15A cells were synchronized as previously described (26). In brief, REY15A cells were arrested at the G2 phase after treatment with 6 mM acetic acid for 6 h. The cell cycle blockage was released through the removal of acetic acid by washing with 0.7% (wt/vol) sucrose twice at 5,000 rpm for 15 min. The cell cycle of synchronized cells was analyzed by flow cytometry using ImageStreamX MarkII Quantitative imaging analysis flow cytometry (Merk Millipore, Germany). The data from the analysis of at least 100,000 cells was collected from each sample and analyzed with the IDEAS data analysis software.

### Western blotting

The expression levels of cell division proteins in the synchronized cell cultures were analyzed by western blot. Around 0.5 × 10^9^ cells were collected at the indicated time points for each sample and run in 12% SDS-PAGE gel, then transferred onto a PVDF membrane. Primary antibodies against ESCRT-III, ESCRT-III-1, and ESCRT-III-2 were produced in rabbits, and a primary antibody against Vps4 was produced in mice by a company (HuaAn Biotechnology Co., Hangzhou, Zhejiang, China). The goat anti-rabbit and goat anti-mouse secondary antibodies (HuaAn Biotechnology Co., Hangzhou, Zhejiang, China) coupled with peroxidase were used as secondary antibodies. The specific bands were detected by chemoluminescence using ECL prime western blotting detection reagents (Amersham) according to the manufacturer’s instructions.

### Immunofluorescence microscopy

*S. islandicus* REY15A cells were collected and pelleted down at 6,000 rpm for 5 min and re-suspended in 300 µL PBS buffer. Then the cells were fixed by the addition of 700 µL cold absolute ethanol and kept at 4°C for at least 2 h. The fixed cells were washed three times with PBST (PBS plus 0.05% Tween-20) to remove the ethanol. Primary antibodies against ESCRT-III, ESCRT-III-1, and ESCRT-III-2 (HuaAn Biotechnology Co., Hangzhou, Zhejiang, China) were added with a dilution of 1:1,000 in PBST and incubated at 4°C overnight. The cells were washed three times and then incubated with the goat anti-rabbit secondary antibody Alexa Fluor 488 (1:1,000, Thermo Fisher Scientific, USA) for the ESCRT-III homologs, and Concanavalin A, Alexa Fluor 647 Conjugate (50 ug/mL, Invitrogen, Thermo Fisher Scientific, USA) for the surface (S-)layer, and kept at 4°C for 2–4 h. The cells were finally washed three times with PBST and resuspended in 70 µL PBS buffer containing 4′,6-diamidino-2-phenylindole (DAPI). A 5 µL of the sample was immobilized with 10 µL mounting media MOWIOL4-88 Reagent (Merk Millipore, Germany) between the slide and the 170 µm-thick coverslip (#1.5) and, after 48 h of polymerization of the mounting media, the localization of ESCRT-IIIs was observed under a Leica SP8 confocal microscope and the data were acquired using the Leica Application Suite X (LAS X) software (Leica).

### Quantification of the fluorescence intensity

The average fluorescence intensities of ESCRT-III, ESCRT-III-1, and ESCRT-III-2 at the “early,” “pre-late,” and “late” stages of membrane constriction during division were measured for 50 cells each. The measurements were made using ImageJ, with a 3 µm by 20 pixel plot profile, and then plotted with the Origin software package.

### Confocal imaging

Samples were imaged on a conventional confocal imaging system with a Leica SP8 laser scanning confocal microscope. This microscope was equipped with a 405 nm laser line and a white light laser, using a Leica HC PL APO CS2 63×/1.40 OIL objective. Pinhole size was set to 1AU. Samples were illuminated with 405, 499/505, 545, and 653 nm laser lines according to the sample labeling. Images with a resolution of 512 × 512 pixels were acquired using PMTs or HyDs detectors. Acquisition parameters were performed at 1,2 µs (dwell time) without line or frame averaging.

### Transmission electron microscopy

For transmission electron microscopy analysis, cell cultures were absorbed into glow-discharged copper grids with carbon-coated form var film and negatively stained with 2.0% (wt/vol) uranyl acetate. The samples were observed under the FEI Tecnai Spirit BioTwin 120 microscope (FEI, Einthoven, The Netherlands) operated at 120 kV.

### Super-resolution imaging with 3D SMLM

#### Microscopy setup

Super-resolution imaging of ESCRT-III proteins was performed on a custom-built 3D SMLM setup based on a Nikon Ti-E Eclipse microscope body (Fig. S6). The setup includes a solid-state laser with an emission wavelength of 640 nm (1 W, 639 nm, Coherent laser Genesis MX STM) and a diode-laser with an emission wavelength of 405 nm (EXLSR-405C-100 mW, Spectra-Physics, Japan). Wavelength selection was done using an optoelectronic device (AA Opto Electronic). The microscope is equipped with a 100× numerical aperture 1.49 oil immersion objective lens, an ultrasensitive electron-multiplying charge-coupled device camera (Andor iXon Ultra 888), and a pair of dual-band-pass dichroic mirrors. For 3D SMLM, the point spread function (PSF) was engineered as a double-helix PSF (43), which was obtained by placing a phase mask provided by the company Double Helix Optics (DH1-670-2045) in a conjugated back focal plane (Fourier Plane) of the microscope (Fig. S6). The spatial phase mask is installed in a 4f system placed in the emission path of the microscope and is composed of two optical achromatic doublet lenses f = 50 mm and f = 75 mm. Together, the lenses allow (i) to cover completely the spatial phase engraved on the mask in order to obtain the same PSF shape in the entire field of view and (ii) to ensure a pixel size of 106 nm, corresponding to the Nyquist criterion. The spatial phase mask was placed in a magnetic holder which can be plugged and unplugged easily and stably. The double-helix PSF was chosen because its depth of imaging (~3 µm) allows to image rings with large diameters (>1 µm), and at different depths.

The analysis and reconstruction of 3D super-resolution images from low-resolution image sequences of single fluorescent molecules was performed with the free ImageJ plugin ZOLA-3D (31), which we have extended for a double helix PSF. First, a z-stack of fluorescent beads is acquired in order to determine the 3D shape of the PSF. These images are used in a calibration step to determine the spatial phase of the entire optical system (spatial phase of the mask and the microscope), and a faithful model of the PSF, which is then used by ZOLA-3D to detect and localize single molecules in 3D.

#### Sample preparation and imaging

The fixed archaea were provided in an Eppendorf tube in PBS. First, 12 mm coverslips were cleaned using a standard protocol optimized for SMLM, whereby the coverslips were plunged successively in acetone, ethanol, and water baths three times and finally plunged in a KOH (1 M) bath under sonication for 1 h. Cleaned coverslips were then rinsed with milliQ water and dried. During this time, a small volume of Poly-L-Lysine (0.1%) mixed with 6 µm diameter beads was prepared (a 1 µL stock solution of beads was diluted in 1 mL of PBS), then 50 µL of this solution was deposited on a cleaned coverslip at room temperature. After 30 min, the poly-L-lysine solution was removed and the coverslips were rinsed with distilled water and dried before putting 50 µL of solution-containing cells on the coverslips. After 30 minutes at room temperature, the excess solution-containing cells was removed. Microscopy slides were layered and sealed with Parafilm. Coverslip-sized holes were then cut into the Parafilm and filled with a photoswitching buffer (0.5 mg/mL glucose oxidase [Sigma], 40 µg/mL catalase [sigma]), 10% wt/vol glucose, and 50 mM of mercaptoethylamine in PBS (pH 7.4). Coverslips were placed inverted onto the Parafilm-coated slides, such that cells faced down into the buffer. Coverslips were sealed with a Parafilm using nail polish prior to imaging.

Using the microscopy setup described above, we acquired sequences of 30,000 widefield fluorescence image frames with a 50 ms exposure time per frame. During acquisition, the 639 nm laser power was kept at a constant intensity, while the 405 nm laser was pulsed with a frequency that was adjusted to ensure that the fraction of fluorescent fluorophores at any given time was low enough to allow the detection and localization of individual Alexa Fluor 647 dyes. Imaging parameters were set using the μManager freeware (http://www.micro-manager.org/) running on a desktop PC and laser control was achieved with custom software.

#### Quantitative analysis of ESCRT-III rings

Quantitative analysis of ring-like structures observed by SMLM was done with a tailor-made Python script. Briefly, for each region of interest defined by the user (corresponding to a single ring, see Fig. 7a; Fig. S8a), the algorithm first fitted a plane to the set of 3D localizations. This was done by minimizing the squared sum of the distances between the plane and the localizations using singular value decomposition. The 3D localizations were then rotated such that the fitted plane coincided with the XY plane (Fig. S7a). Finally, a closed 3D curve modeled as a high-order trigonometric polynomial ring was fitted to the localizations using a least squares optimization function from a numpy linear algebra package (Fig. S7b). From this fitted curve, we computed the circumference and the thickness, where the latter was defined as the variance of the distance between the 3D localizations and the fitted polynomial curve.

To validate this automated measurement procedure, we generated simulated 3D SMLM data for ring-like shapes of various sizes, thicknesses, and orientations (Fig. S7) with and without accounting for random localization errors and the size of antibody labels. Specifically, we simulated closed ring-like curves with circumferences ranging from 0.5 µm to 5 µm, oriented randomly in 3D. In order to account for deviations from perfect circularity and planarity, we defined the curves using high-order trigonometric polynomials. For each simulated ring-like structure, we generated *n* = 1,000 localizations (close to the average number of localizations in experiments) and displaced them in a random 3D direction by a distance of 17.5 nm to account for the size of the antibody labels and the resulting distance between the fluorophore and labeled protein. We then added isotropic normally distributed random localization errors with a standard deviation (precision) of 25 nm along each axis, based on estimates of the Cramer-Rao error bounds from the experimental data computed by ZOLA-3D (Fig. S7e).

We then compared the measured thickness on the simulated structures to the ground truth thickness for a wide range of circumferences (Fig. S7c and d). In the absence of localization errors (precision 0 nm), the measured thickness was in excellent agreement with the ground truth thickness regardless of the assumed circumference (Fig. S7d, gray dots). In the presence of localization errors (precision 25 nm), the measured thickness was overestimated for ring thicknesses below ~100 nm and was ~40 nm for zero-thickness rings, thereby defining a resolution limit for measuring ring thickness with our technique (Fig. S7d, blue dots). This simulation analysis suggests that our measurements are insensitive to changes in ring thicknesses for thicknesses below ~40 nm but can distinguish the thickness of different rings as long as at least one ring is thicker than about ~40 nm.

## SUPPLEMENTAL MATERIAL

Supplemental material mbio.00991-24-s0001.pdf Supplemental figures and tables.

Video S1a mbio.00991-24-s0002.avi An example of an animated 3D view of ESCRT-III structures imaged in super-resolution with 3D SMLM.

Video S1b mbio.00991-24-s0003.avi An example of an animated 3D view of ESCRT-III structures imaged in super-resolution with 3D SMLM.

Video S1c mbio.00991-24-s0004.avi An example of an animated 3D view of ESCRT-III structures imaged in super-resolution with 3D SMLM.

Video S1d mbio.00991-24-s0005.avi An example of an animated 3D view of ESCRT-III structures imaged in super-resolution with 3D SMLM.

ASM does not own the copyrights to Supplemental Material that may be linked to, or accessed through, an article. The authors have granted ASM a non-exclusive, world-wide license to publish the Supplemental Material files. Please contact the corresponding author directly for reuse.
