# Supplementary material for: A relay race of ESCRT-III paralogs drives cell division in a hyperthermophilic archaeon
Source: mBio. 2024 Dec 19;16(2):e00991-24. doi: 10.1128/mbio.00991-24 (PMC11796394; doi:10.1128/mbio.00991-24)
Supplement: Supplemental material — Supplemental figures and tables. [file mbio.00991-24-s0001.pdf]

SUPPLEMENTARY INFORMATION

Supplementary figures

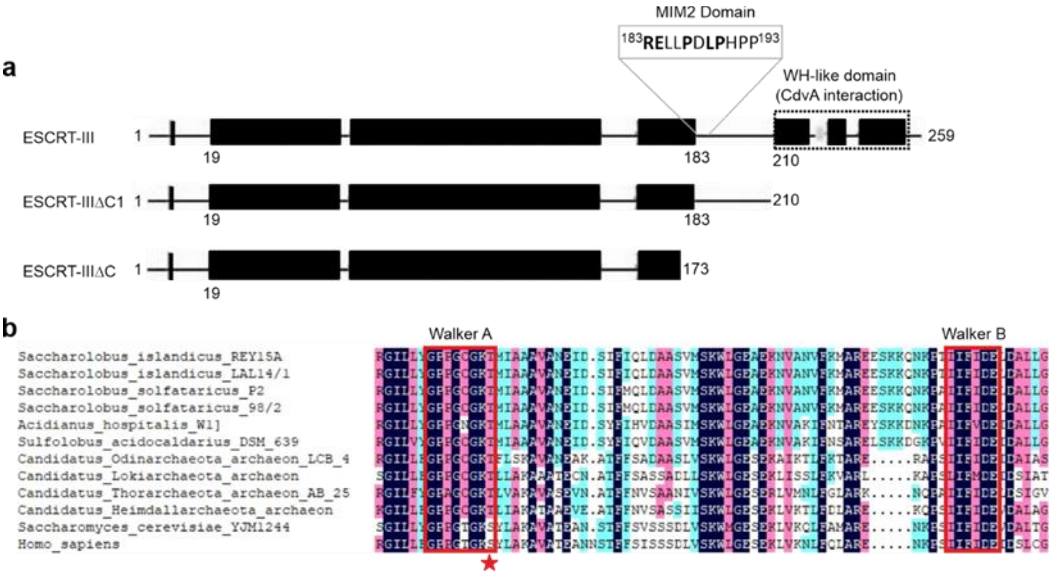

Figure S1. Construction of the dominant-negative mutants of ESCRT-III and Vps4 proteins. **a.** Secondary structure and domain organization of ESCRT-III. ESCRT-III has the C-terminal winged helix (WH)-like domain that interacts with CdvA. In ESCRT-IIIΔC1, the C-terminal WH-like domain is removed, but the MIM2 domain responsible for interaction with Vps4 is retained. Note that ESCRT-IIIΔC1 constructed in this study is different from the previously reported ESCRT-IIIΔC, which lacked both WH and MIM2 domains (1). **b.** Sequence alignment of the AAA+ ATPase Vps4 homologs from diverse archaeal and eukaryotic species. The conserved Walker A and Walker B motifs are important for the ATP binding and hydrolysis activities, respectively. In the dominant-negative Vps4-T148A mutant Thr148 residue (red star) located in the ATP-binding loop of the Walker A motif has been substituted with an Ala.

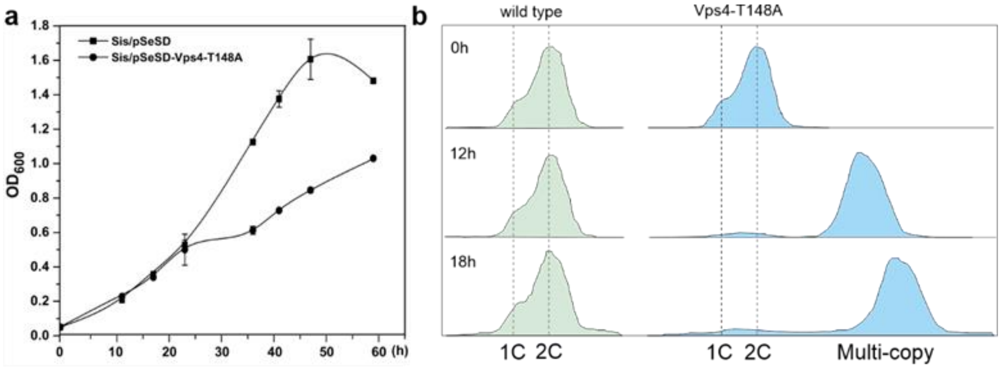

Figure S2. Over-expression of the dominant-negative Vps4-T148A mutant causes growth retardation and cell division deficiency. **a-b.** Growth curves (a) and flow cytometry analysis (b) of cells over-expressing the Vps4 mutant. Cells harboring the empty vector pSeSD were used as a control. Cells were collected at the indicated time points and analyzed for the DNA content by flow cytometry. Note that after ~50 h of incubation, control *S. islandicus* cells enter the “death phase”, as described previously (2), but the mechanism underlying this phenomenon remains unclear.

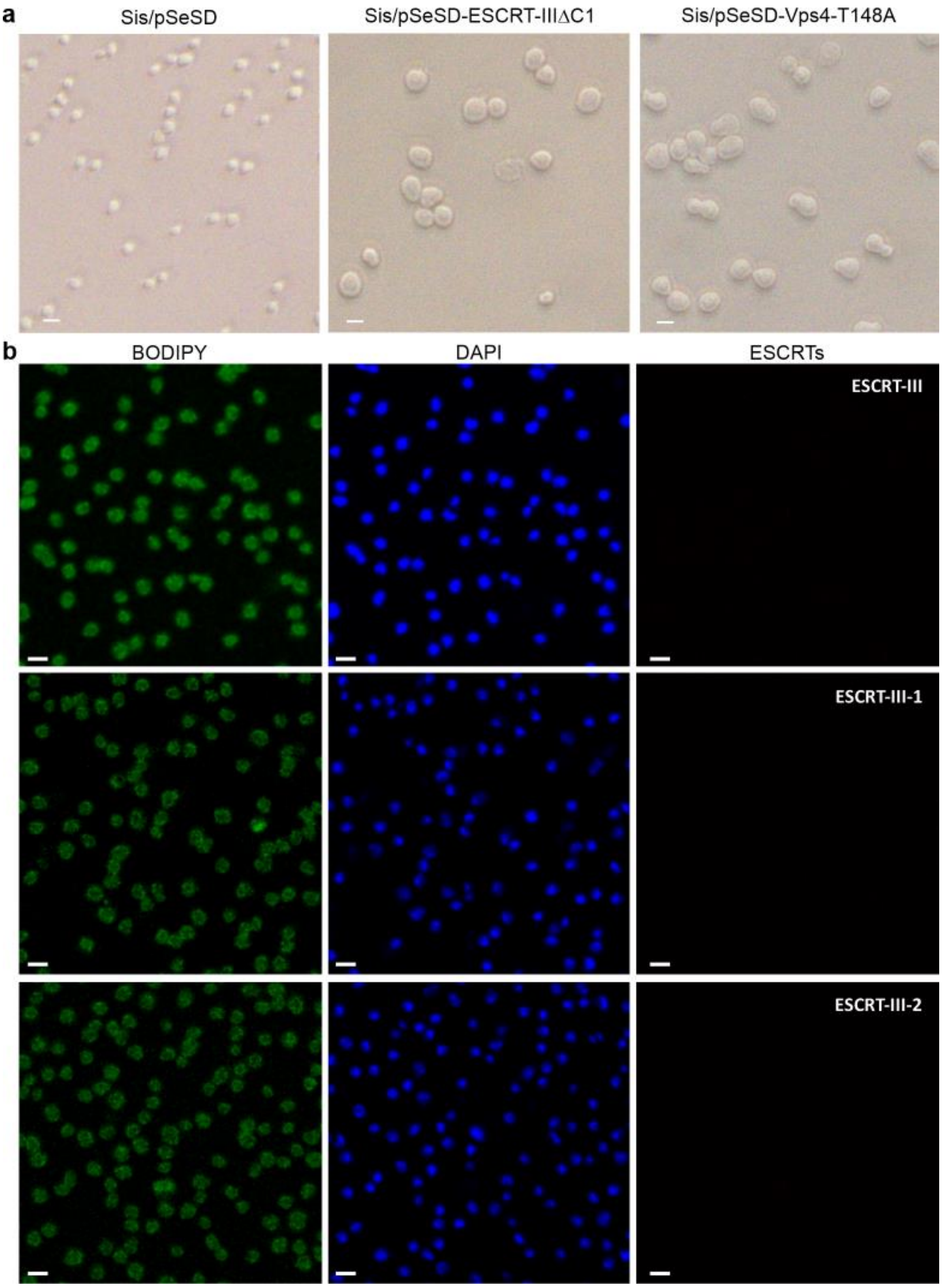

Figure S3. The interaction between ESCRT-III and CdvA is necessary for the ESCRT-III ring formation. **a.** Bright-field microscopy of *S. islandicus* cells containing the empty vector pSeSD (control; left) or overexpressing the dominant-negative ESCRT-IIIΔC1 (middle) or Vps4 (right) mutants for 12h. **b.** Sub-cellular localization of the three ESCRT-III paralogs (indicated in the top right corner of each row) in cells over-expressing the dominant-negative ESCRT-IIIΔC1 mutant in which the CdvA-interacting WH domain has been deleted. The cells were collected at 5 h after induction by arabinose and subjected to the immunofluorescence microscopy analysis. Scale bars, 2 μm.

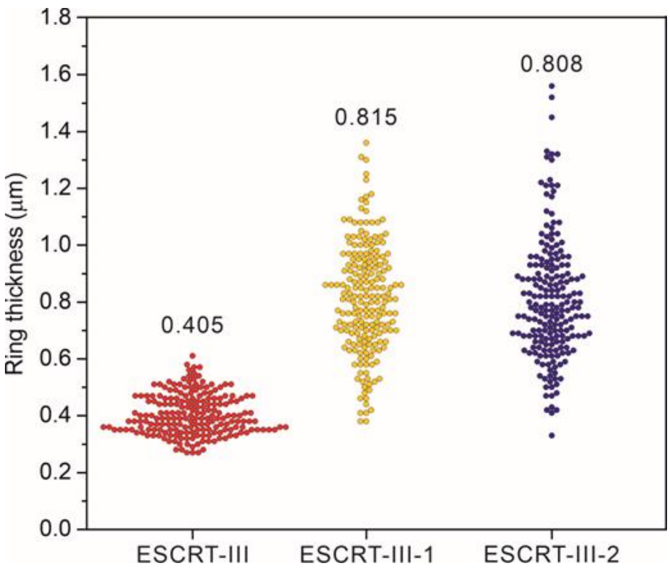

Figure S4. Thickness of the rings formed by ESCRT-III homologs in the Sis/pSeSD-Vps4-T148A strain and measured from confocal microscopy images. The cells were synchronized with acetic acid treatment. The dominant negative Vps4 mutant was induced by arabinose for 5 h. For each of the three ESCRT-III homologs, 200 cells were randomly selected and the thickness of the corresponding division rings was measured using the LAS X (Leica) software. The average thickness of the division rings is indicated on top of the corresponding violin plot.

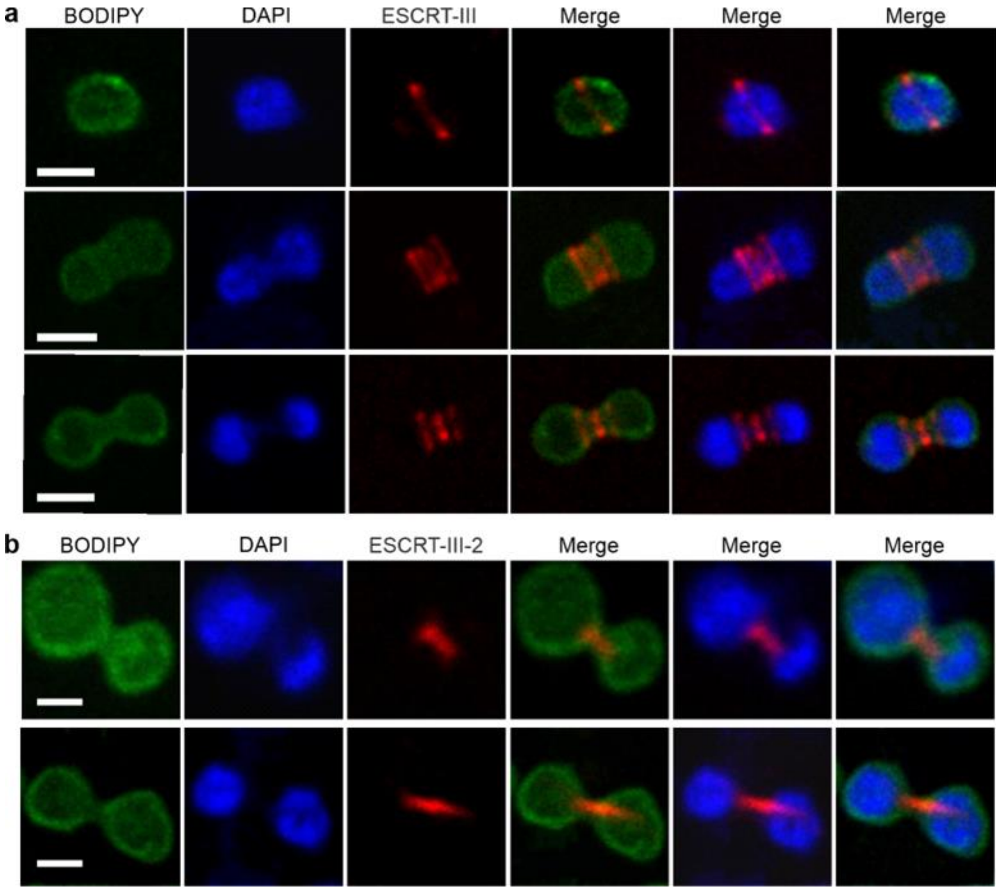

Figure S5. Sub-cellular localization of ESCRT-III and ESCRT-III-2 in cells over-expressing the dominant-negative Vps4 mutant for 12h. **a.** After prolonged overexpression of the Vps4 mutant, multiple ESCRT-III rings could be observed. **b.** Fluorescent signal corresponding to ESCRT-III-2 was distributed along the membrane bridge. BODIPY was used to stain the membrane, whereas the nucleoids were stained with DAPI (4’,6-diamidino-2-phenylindole). ESCRT-III and ESCRT-III-2 were visualized using fluorescently-labelled antibodies. Scale bars, 2 µm.

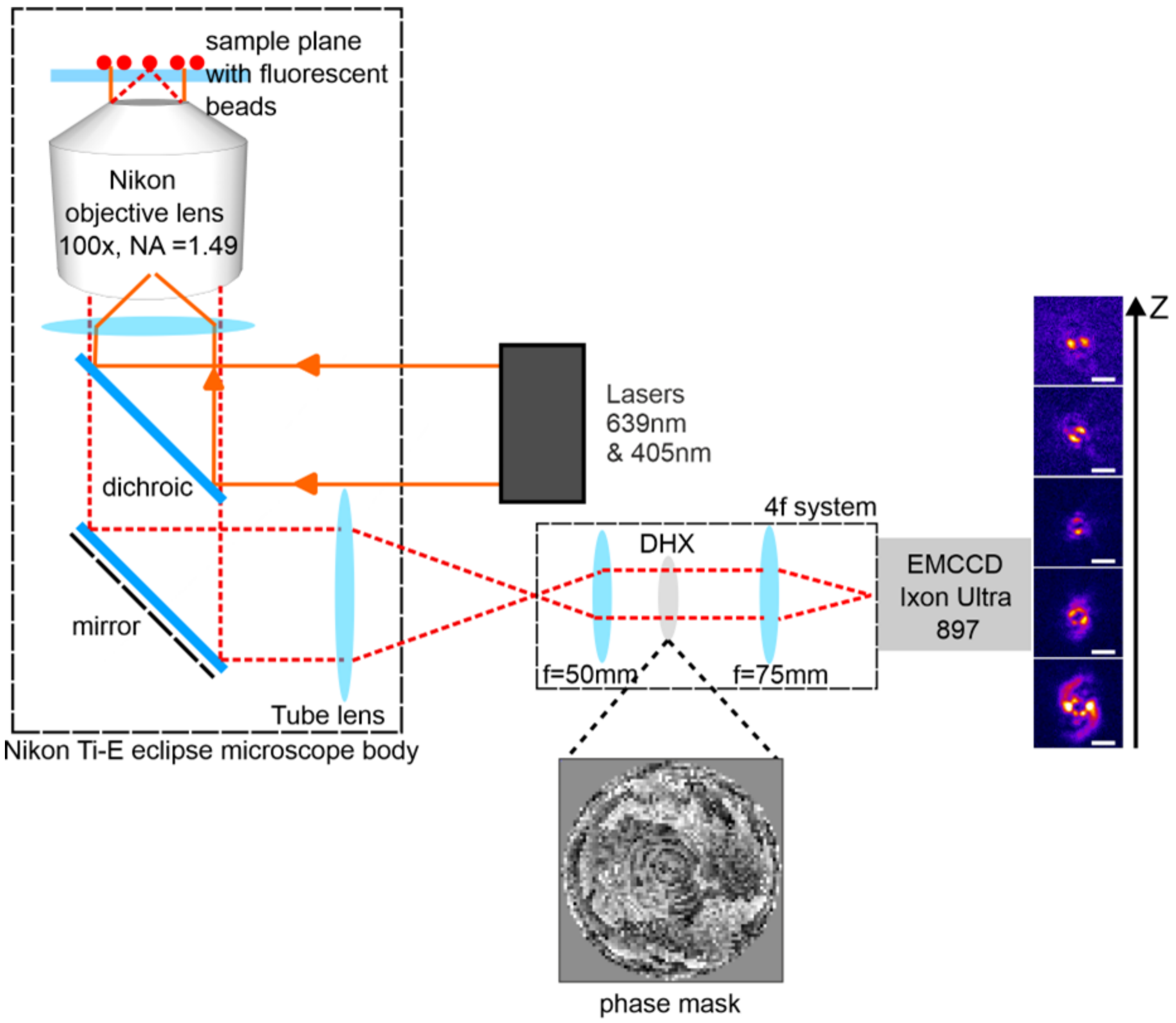

Figure S6. Scheme of the custom-built 3D SMLM system. Two lasers (639nm & 405nm) are focused in the back focal plane of the 100x NA1.49 objective lens of a Nikon Ti-E eclipse microscope. The image is acquired with an EMCCD camera (Andor Ixon Ultra 897). The emission path features a 4f system with a spatial phase mask (DHX, DH1-670-2045) in the Fourier Plane. Because of this phase mask, the 3D image of a point-like fluorescent object (point spread function, PSF) resembles a double helix, as seen in the z-stack of a fluorescent bead shown on the right. The spatial intensity pattern in a single 2D plane encodes the axial (z) coordinate. The ZOLA-3D software (https://github.com/imodpasteur/ZOLA-3D) calibrates a PSF model using a z-stack of fluorescent beads (Tetraspeck) spread over the field of view and uses this model to reconstruct 3D super-resolution images from low resolution 2D image sequences.

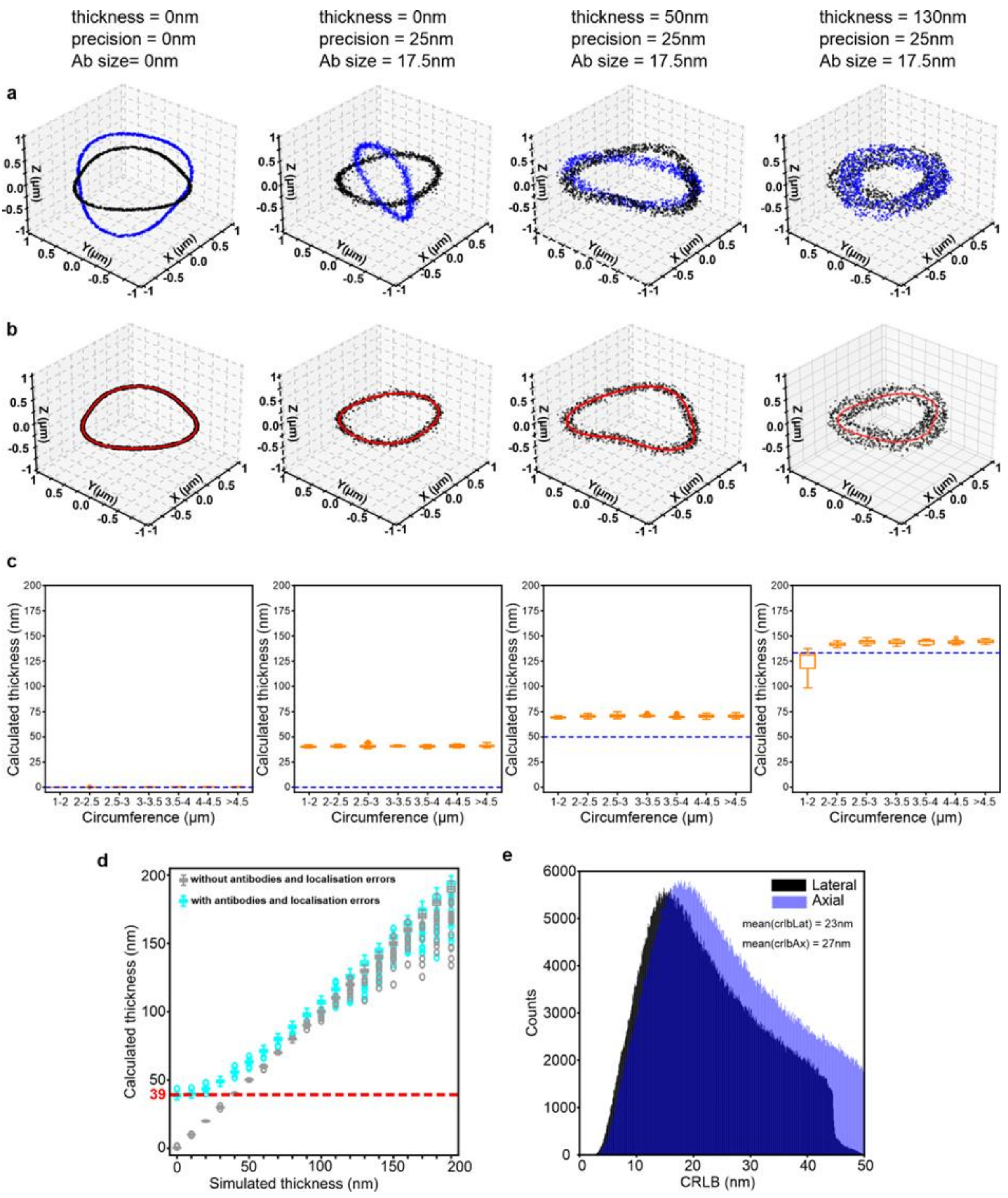

Figure S7. Simulations validate calculation of ring thickness. **a.** Black dots show simulated SMLM data for 3D ring-like structures with different orientations, circumferences and thicknesses, with (columns 2-4) or without (column 1) random localization errors of standard deviation (precision) 25 nm (see panel e) and with (columns 2-4) or without (column 1) antibody labels of 17.5 nm size. Blue dots show the localizations after rotation towards the XY plane. **b.** A closed curve (red) is fitted to these rotated localizations. **c.** Box plots show the calculated ring thickness from the simulated data corresponding to each column for different ranges of circumferences. Blue dotted lines show the true thickness. **d.** Scatter plot shows the calculated vs. true thickness from simulations with or without localization errors (25 nm precision) and 17.5 nm size antibody labels. The red dotted line shows the minimal thickness that can be resolved by our method. **e.** Histograms show the lateral (black) and axial (blue) Cramer Rao Lower Bounds (CRLB) (which measure the theoretically achievable localization precision) computed by ZOLA-3D on the experimental data. The mean CRLBs is the basis for our assumption of a 25 nm localization precision in simulated data.

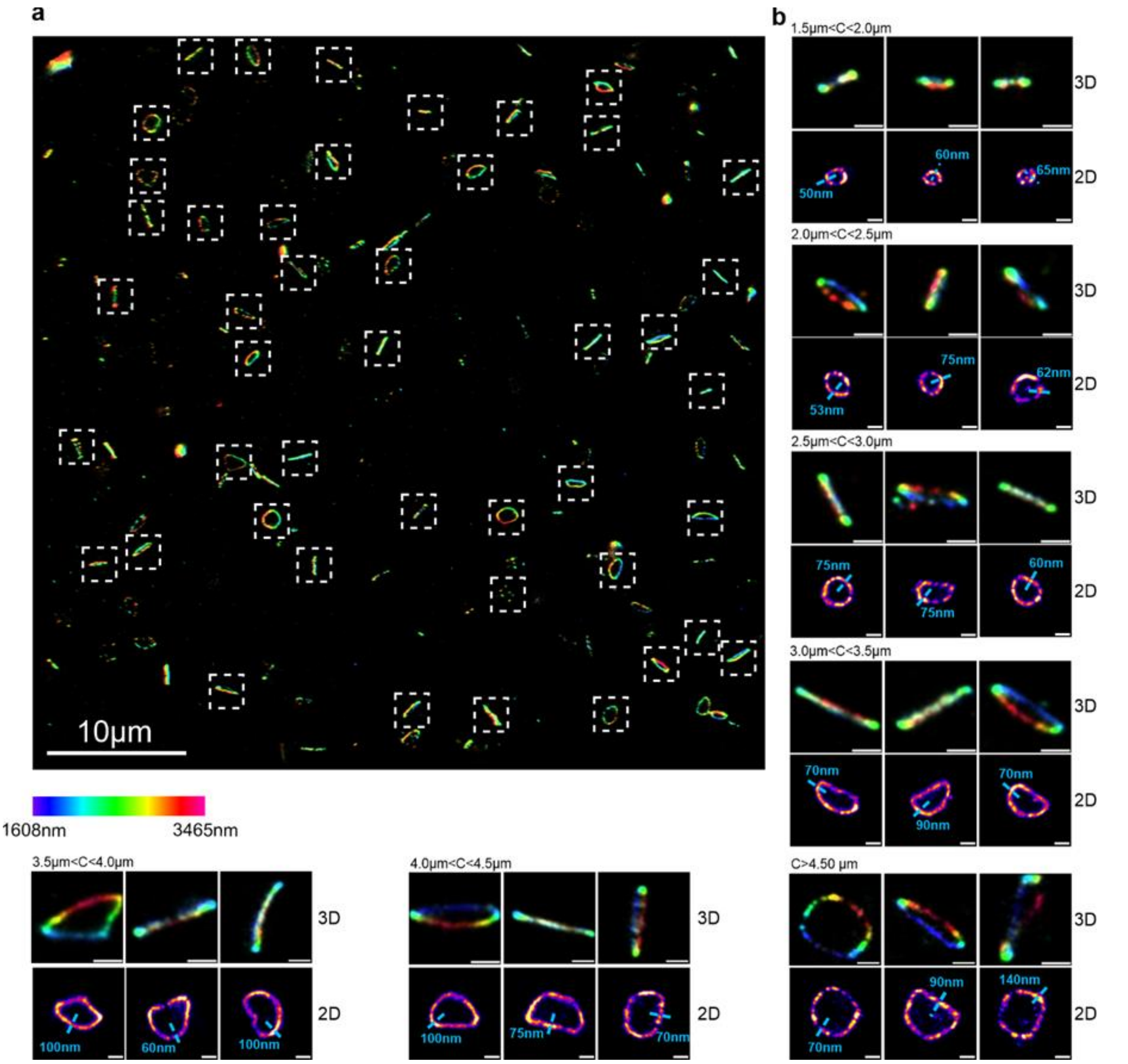

Figure S8. Super-resolution characterization of ESCRT-III rings. **a.** Three-dimensional SMLM super-resolution image of ESCRT-III-labeled *S. islandicus* cells. An entire field of view, containing tens of individual cells, is shown. The color encodes axial coordinates (see color bar). Dashed yellow boxes indicate cells selected manually for further analysis. **b.** Color-coded 3D views (top rows) and rotated 2D views of ESCRT-III rings (bottom rows) are shown. Scale bars, 250 nm. The thickness of each ring was measured at manually selected locations (dashed blue lines) as indicated.

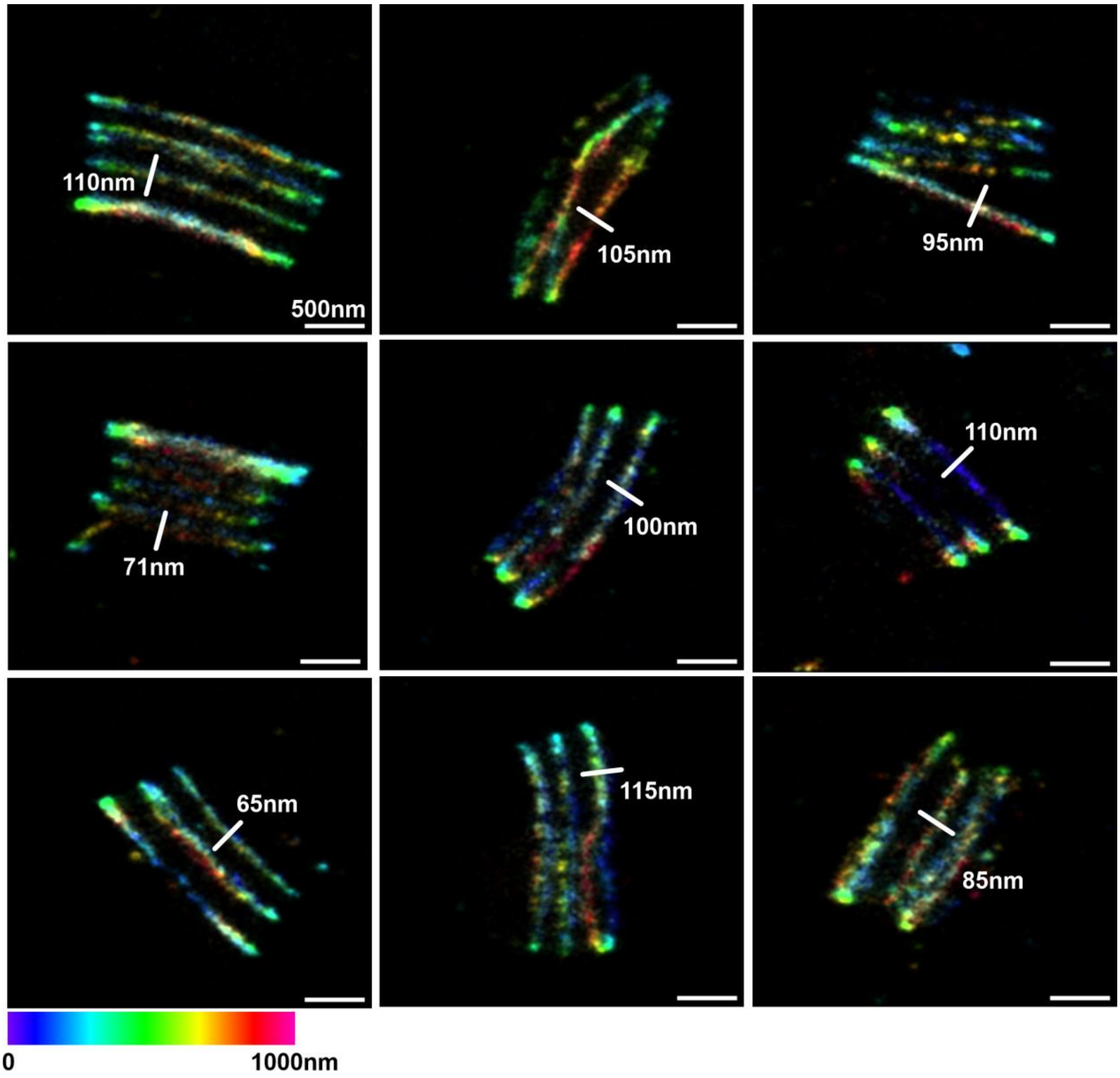

Figure S9. Three-dimensional super-resolution SMLM image of ESCRT-III rings in cells over-expressing the dominant-negative Vps4 mutant for 12h. Color scale encodes axial coordinates (see color bar). Manual measurements of ring thickness from intensity profiles at selected locations (white bars) are indicated. Supplementary data file 1 includes four videos showing the ESCRT-III rings in 3D (Supplementary data file 1).

Supplementary tables

Table S1. Plasmids used in this study.

Plasmid	Description	Reference
pSeSD	Empty vector	
pSeSD-ESCRT-III-1ΔC	ESCRT-III-1ΔC overexpression	(1)
pSeSD-ESCRT-III-2ΔC	ESCRT-III-2ΔC overexpression	(1)
pSeSD-ESCRT-IIIΔC1	ESCRT-III ΔC1 overexpression	This study
pSeSD- Vps4-T148A	Vps4-T148A overexpression	This study
pGE-ESCRT-III-1	*escrt-III-1*deletion	This study
pGE-ESCRT-III-2	*escrt-III-2* knockdown	This study

Table S2. *Saccharolobus islandicus* strains used in this study.

Strain	Phenotype	Reference
Sis/pSeSD	Control	
Sis/pSeSD-ESCRT-III-1ΔC	ESCRT-III-1ΔC overexpression	(1)
Sis/pSeSD-ESCRT-III-2ΔC	ESCRT-III-2ΔC overexpression	(1)
Sis/pSeSD-ESCRT-IIIΔC1	ESCRT-III ΔC1 overexpression	This study
Sis/pSeSD-Vps4-T148A	Vps4-T148A overexpression	This study
Δescrt-III-1/pSeSD	*escrt-III-1*deletion	This study
Δescrt-III-1/pSeSD-Vps4-T148A	*escrt-III-1*deletion and Vps4-T148A over-expression	This study
Δescrt-III-1/pGE-ESCRT-III-2	*escrt-III-1*deletion and *escrt-III-2* knockdown	This study

Table S3. Oligonucleotides used in this study.

Oligonucleotide name	Sequence
escrt-III-1-spacer-F	AAGACAACTCCTAATACTGGGATTAAGTTTACAAATACATCTC
escrt-III-1-spacer-R	AGCGAGATGTATTTGTAAACTTAATCCCAGTATTAGGAGTTGT
escrt-III-1-L-arm-F-SphI	AAGTACAATTGTGCTGCATGCTCTGGTCCAATGTATATTCT
escrt-III-1-L-arm-R	AGGTAAGTTATATATTAGCCTGAAGGAGAAGTTCCCAGAC
escrt-III-1-R-arm-F	GTCTGGGAACTTCTCCTTCAGGCTAATATATAACTTACCT
escrt-III-1-R-arm-R-XhoI	TTAACATATTGGATGCTCGAGATCCTGTATCGTTCTCTAAA
escrt-III-2-Spacer-F	AAGTCCCCTGCTTCTATTACTACCTCTTGTAATCCCTCCTCTA
escrt-III-2-Spacer-R	AGCTAGAGGAGGGATTACAAGAGGTAGTAATAGAAGCAGGGGA
vps4-F-NdeI	ATGCCATATGAGTGCTCAAGTAATGCTAG
vps4-R-SalI	TCACGTCGACTAATGCCTTAAACTTCTCT
vps4-T148A-F	CCAGGTTGTGGTAAACGTATGATAGCTG
vps4-T148A-R	CAGCTATCATACGTTTACCACAACCTGG

Supplementary data files

Supplementary data file 1 includes four videos (Video S1a-d) which are examples of ESCRT-III structures imaged in super-resolution with 3D SMLM.

Supplementary references

1. Liu J, Gao R, Li C, Ni J, Yang Z, Zhang Q, Chen H, Shen Y. 2017. Functional assignment of multiple ESCRT-III homologs in cell division and budding in Sulfolobus islandicus. Mol Microbiol 105:540-553.
2. Liu J, Cvirkaite-Krupovic V, Commere PH, Yang Y, Zhou F, Forterre P, Shen Y, Krupovic M. 2021. Archaeal extracellular vesicles are produced in an ESCRT-dependent manner and promote gene transfer and nutrient cycling in extreme environments. ISME J 15:2892-2905.
